# In-situ TiO_2-x_ decoration of titanium carbide MXene for photo/sono-responsive antitumor theranostics

**DOI:** 10.1186/s12951-022-01253-8

**Published:** 2022-01-28

**Authors:** Dong-Yang Zhang, Hengke Liu, Muhammad Rizwan Younis, Shan Lei, Yunzhi Chen, Peng Huang, Jing Lin

**Affiliations:** 1 Department of Hepatobiliary Surgery, The First Affiliated Hospital of Wenzhou Medical University Wenzhou, Zhejiang, 325000 China; 2 International Cancer Center, Laboratory of Evolutionary Theranostics (LET), School of Biomedical Engineering, Marshall Laboratory of Biomedical Engineering, Shenzhen University Health Science Center, Shenzhen, 518060 China

**Keywords:** Titanium carbide, Oxygen deficient titanium dioxide, Photoacoustic imaging, Photothermal therapy, Sonodynamic therapy

## Abstract

**Background:**

Sonodynamic therapy (SDT) has emerged as a noninvasive therapeutic modality that involves sonosensitizers and low-intensity ultrasound. However, owing to the rapid recombination of charge carriers, most of the sonosensitizers triggered poor reactive oxygen species (ROS) generation, resulting in unsatisfactory sonodynamic therapeutic effects.

**Results:**

Herein, a photo/sono-responsive nanoplatform was developed through the *in-situ* systhesis of TiO_2-x_ on the surface of two-dimensional MXene (titanium carbide, Ti_3_C_2_) for photoacoustic/photothermal bimodal imaging-guided near-infrared II (NIR-II) photothermal enhanced SDT of tumor. Because of several oxygen vacancies and smaller size (~ 10 nm), the *in-situ* formed TiO_2-x_ nanoparticles possessed narrow band gap (2.65 eV) and high surface area, and thus served as a charge trap to restrict charge recombination under ultrasound (US) activation, resulting in enhanced sonodynamic ROS generation. Moreover, Ti_3_C_2_ nanosheets induced extensive localized hyperthermia relieves tumor hypoxia by accelerating intratumoral blood flow and tumor oxygenation, and thus further strengthened the efficacy of SDT. Upon US/NIR-II laser dual-stimuli, Ti_3_C_2_@TiO_2-x_ nanoplatform triggered substantial cellular killing in vitro and complete tumor eradication in vivo, without any tumor recurrence and systemic toxicity.

**Conclusion:**

Our work presents the promising design of photo/sono-responsive nanoplatform for cancer nanotheranostics.

**Graphical Abstract:**

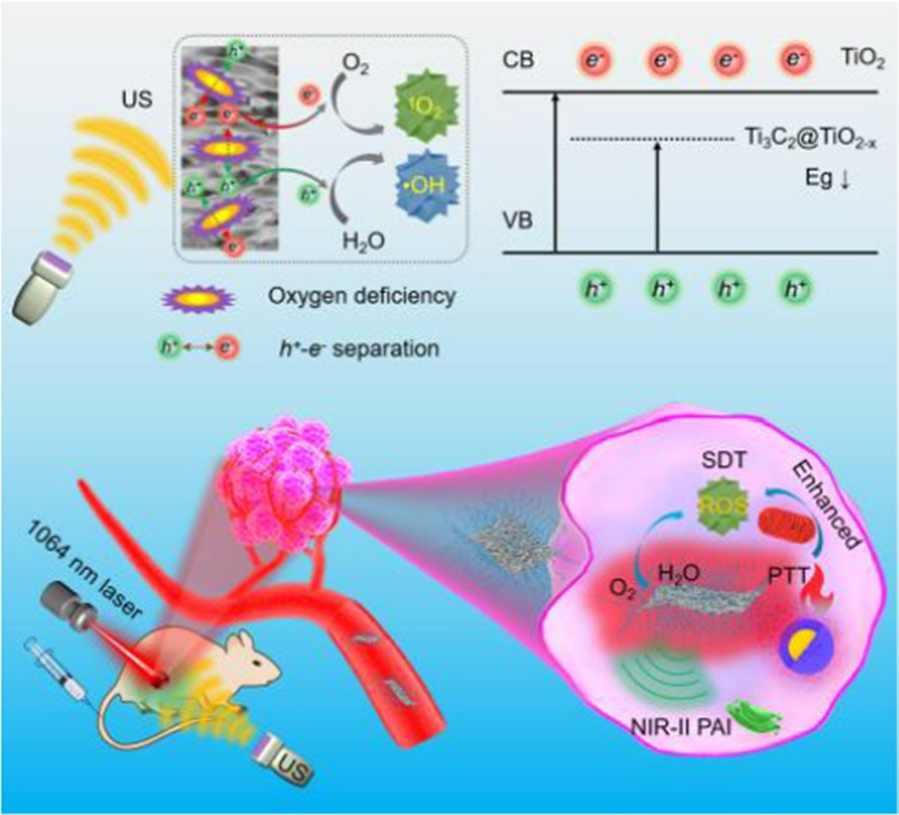

**Supplementary Information:**

The online version contains supplementary material available at 10.1186/s12951-022-01253-8.

## Introduction

Sonodynamic therapy (SDT), a type of non-invasive tumor modality, offered promising advantages than conventional tumor treatment, such as high tissue penetration, high spatiotemporal selectivity, and non-invasiveness [1–6]. In SDT, sonosensitizers triggered reactive oxygen species (ROS) production under ultrasound (US) stimulation, leading to selective tumor cell killing with minimal damage to nearby healthy cells [7–11]. Hence, several organic and inorganic sonosensitizers have been developed, however, organic sonosensitizers are mainly suffer from high skin phototoxicity [12, 13]. Alternatively, inorganic sonosensitizers with negligible phototoxicity and enhanced chemical stability, respectively, have shown great promise for tumor SDT. Especially, titanium dioxide (TiO_2_) as an inorganic sonosensitizer, has been widely employed, but the rapid recombination (50 ± 30 ns) of US-mediated charge carriers (electron–hole pair) endows TiO_2_ with poor ROS quantum yield, resulting in limited SDT efficacy [14]. Previous reports suggested that the presence of oxygen deficiencies within TiO_2_ or the integration of TiO_2_ with noble metals could improve the SDT activity [15–18], however, because of the large size TiO_2_ NPs, the adsorption of oxygen molecules onto the surface of TiO_2_ is remarkably poor, leading to unsatisfactory ROS yield. Thus, an optimal design engineering of inorganic sonosensitizers is desirable to overcome these limitations, promoting high SDT performance under US activation. Moreover, as the SDT reaction is exclusively dependent on tissues oxygen, the continuous sonodynamic ROS generation induced severe tumor hypoxia due to oxygen depletion, and hence, restricts the overall SDT efficacy. Therefore, the integration of SDT with other non-invasive tumor treatment modalities, which may complement SDT effects, is greatly needed.

Being activated by an external light source, photothermal therapy (PTT) is an ideal non-invasive treatment modality, which induced irreversible destruction of tumor cells via localized hyperthermia generated by photothermal agents [19–22]. As photothermal agents with NIR-I light absorption have been actively developed, NIR-I tumor PTT is well-established, while PTT of deep-seated tumors at second biowindow (NIR-II) is yet limited. Moreover, though NIR-I laser light excitation endows PTT with sufficient penetration depth, an inhomogeneous thermal distribution as well as tumor self-regulation limit the complete photothermal eradication of deep-seated tumors, favoring tumor recurrence and metastasis [23]. Previously, Xia et al. demonstrated photothermal enhanced photodynamic therapy (PDT) of Hela tumor in vivo under low power single NIR-I laser activation [24]. They suggested that localized hyperthermia could not only activate PDT by the controlled release of indocyanine green, but also relieve PDT-induced tumor hypoxia, leading to accelerate photodynamic tumor killing. Recently, our group have shown that PTT under both NIR-I and NIR-II laser excitation, could enhance multimodal tumor therapies such as starvation therapy, chemodynamic therapy, and immunotherapy [25–28]. Notably, it has been reported that PTT could complement SDT as mild hyperthermia accelerates intratumoral blood flow and tumor oxygenation, which promote sonodynamic ROS generation by relieving tumor hypoxia [29–31]. Whereas, under US activation, SDT also complements PTT by ROS-mediated tumor cell killing as well as replenishment of thermally resistant deeply localized tumors due to the high penetration depth of US [32]. Considering the prominent features of individual PTT and SDT, their integration would be highly advantageous to overcome their inherent limitations and achieve ultimate therapeutic effects.

**Scheme 1:**
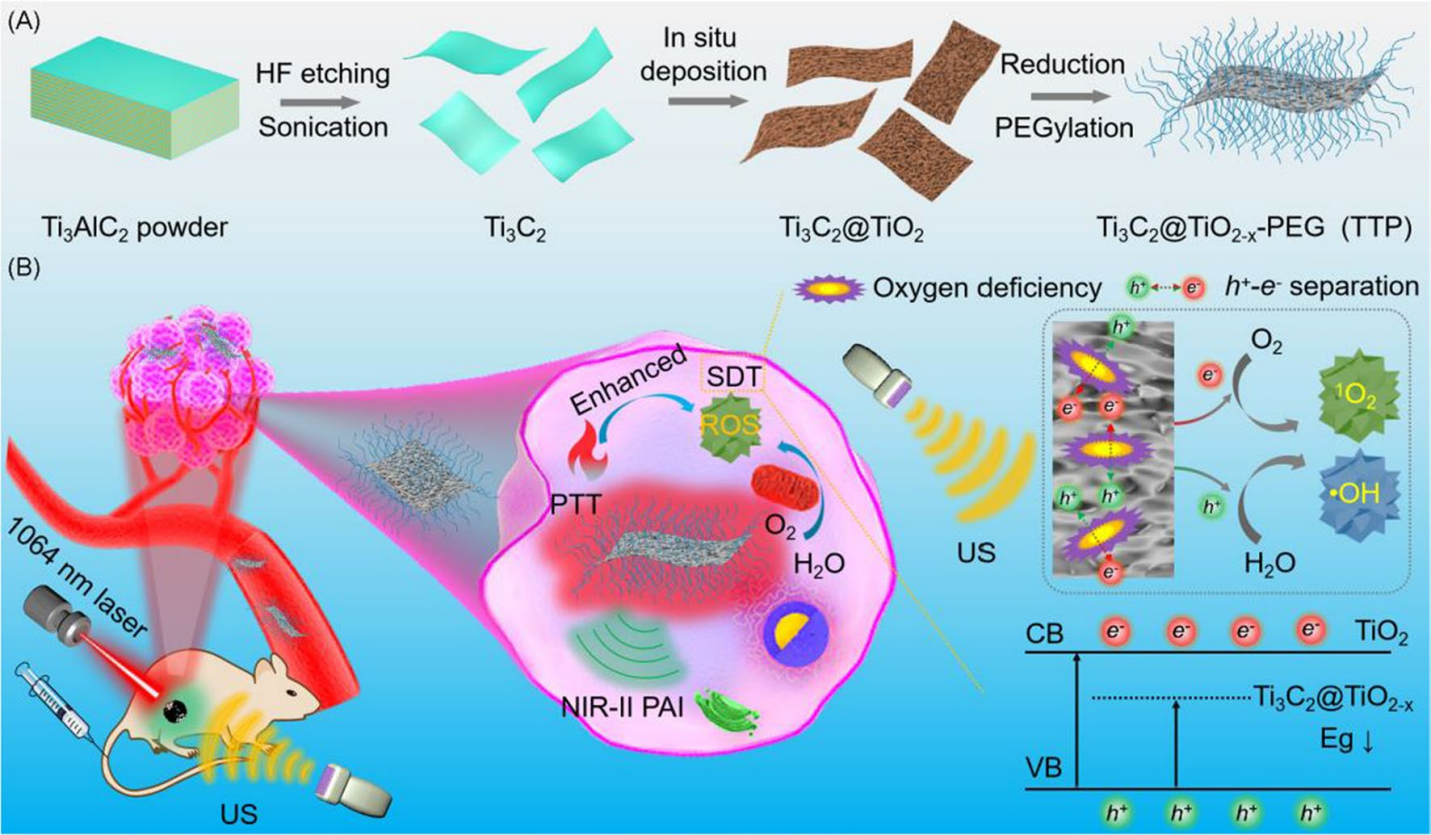
Schematic illustration of **A** the preparation of *in-situ* fabricated TTP nanohybrids and **B** their application in bimodal PA/PT imaging guided synergistic photothermal-enhanced sonodynamic therapy in NIR-II biowindow

Two-dimensional MXene nanomaterials, such as titanium carbide (Ti_3_C_2_), are widely used in biomedical applications [33–40]. Particularly, Ti_3_C_2_ with good absorption in NIR-II region, excellent photothermal performance, and low toxicity, has become a potential photothermal therapeutic agent. Furthermore, multimodal imaging capacity of Ti_3_C_2_ provides an opportunity to monitor nanomaterials biodistribution as well as effectively guide the tumor treatment in vivo. Owing to the promising biomedical characteristics, Ti_3_C_2_ is an ideal candidate to couple with sonosensitizer to trigger PTT enhanced SDT of deep-seated tumors at second biowindow. In this study, a Ti_3_C_2_-based 2D nanotheranostic hybrid is constructed *in-situ* for duplex photoacoustic (PA)/photothermal imaging-guided synergistic PTT/enhanced SDT at NIR-II biowindow (Scheme 1). In brief, the multifunctional nanohybrid (Ti_3_C_2_@TiO_2-x_) is developed by a hydrothermal method via* in-situ* growth of TiO_2-x_ nanoparticles with several oxygen defects onto Ti_3_C_2_ nanosheets. The resultant Ti_3_C_2_@TiO_2-x_ were further modified with polyethylene glycol (PEG), defined as TTP, showing good biocompatibility and aqueous dispersibility in various physiological solutions. The as-obtained TTP not only exhibits good photothermal performance, but also demonstrates superior ROS production under US excitation than TiO_2_ due to high surface area and the presence of multiple oxygen vacancies, promoting enhanced separation of charge carriers (electron–hole pairs). A remarkably higher therapeutic efficacy was achieved under dual-modal PA/PT imaging guidance both in vitro and in vivo, without any obvious toxic effects after intravenous administration. This study provides a promising strategy of photo-sonoinduced theranostics of deeply localized tumors.

## Material and methods

### Preparation of Ti3C2@TiO2-x-PEG (TTP)

Ti_3_C_2_ nanosheets were obtained following the reported literature [36]. Then, 2D Ti_3_C_2_@TiO_2_ hybrid was fabricated according to the previous report with slight modification. 100 mg of Ti_3_C_2_ was dispersed in 3.0 M hydrochloric acid (15 mL) containing 0.04 M ammonium fluoride. Above dispersion was transferred to the Teflon stainless steel autoclave and kept at 200 °C for 12 h. After centrifugation and washing with deionized (DI) water, the as-collected Ti_3_C_2_@TiO_2_ precipitate was added to hydrazine hydrate solution (25 mL, 50 wt%) under stirring. Next, the suspension was further transferred to the Teflon stainless steel autoclave and kept at 200 °C for 12 h. The final Ti_3_C_2_@TiO_2-x_ hybrid was obtained by centrifugation and washing with DI water.

To improve the solubility and biocompatibility of Ti_3_C_2_@TiO_2-x_, 20 mg of DSPE-PEG dissolved in chloroform (20 mL) was added dropwise into Ti_3_C_2_@TiO_2-x_ ethanol solution (2 mg/mL, 20 mL) under ultrasonication. The product was obtained by evaporating the solvent and then resuspended in phosphate buffered saline (PBS), and finally stored at 4 °C for future use.

### In vivo toxicity

BALB/c female mice (n ≥ 3) were intravenously (*i.v.*) injected with TTP nanohybrids (4 mg/mL, 200 μL) or PBS (200 μL). The body weight of mice was recorded every 3 days. The blood and primary organs (kidneys, lungs, spleen, liver, heart) were acquired after 30 days post-injection. The blood was used to perform hematologic analysis and the primary organs were stained by hematoxylin and eosin (H&E). All animal experiments were conducted as per the approved institutional guidelines for the care of laboratory animals of Shenzhen University.

### Photoacoustic/thermal imaging and biodistribution of nanohybrids in vivo

PA signals of the varying concentrations of TTP nanohybrid in solution were recorded by a photoacoustic imager. For in vivo NIR-II PA imaging, 4T1 tumor-bearing mice (n = 3) were *i.v.* injected with TTP nanohybrids (2 mg/mL, 200 μL), and the PA signals were monitored at different time points (0, 1, 2, 4, 8, and 24 h).

4T1 tumor-bearing BALB/c nude mice were *i.v.* injected with TTP nanohybrid (2 mg/mL, 200 μL). After 4 h post-injection, the tumors were irradiated by a 1064 nm laser and the real-time temperature changes were monitored with a thermal imager.

4T1 tumor-bearing nude mice (n = 3) were *i.v.* injected with TTP nanohybrid (2 mg/mL, 200 μL). After 24 h post-injection, the mice were euthanized to collect the primary organs. The primary organs were digested by aqua regia and determined by ICP-MS. The biodistribution of Ti (% ID of Ti per gram of tissues) was calculated in primary organs.

### In vivo treatment

4T1 tumor-bearing BALB/c nude mice with ~ 50 mm^3^ tumor volume, were randomly divided into 6 groups (n ≥ 5) as follows: (i) PBS, (ii) laser + US (iii) TTP nanohybrid, (iv) TTP nanohybrid + US, (v) TTP nanohybrid + laser, (vi) TTP nanohybrid + laser + US. After 4 h post-injection, the tumors were treated with laser irradiation (1064 nm, 0.8 W/cm^2^, 10 min) or/and US (1 W/cm^2^, 5 min). The tumor volumes and body weights of mice were recorded every 2 days. The tumor volume was calculated based on the formula: V (mm^3^) = AB^2^/2, where A and B are the maximum length (mm) and the minimum width (mm) of the tumor, respectively. The body weights of mice were recorded every 2 days. The mice were sacrificed at 14th day post-treatment to collect tumors, serum, and primary organs. The tumors were photographed, weighed, and stained by Terminal-deoxynucleoitidyl transferase mediated nick end labeling (TUNEL) assay. The serum was used to determine the levels of liver and kidney function indicators, while the primary organs and tumor were stained by H&E for histopathological investigations.

## Results and discussion

### Synthesis and characterization

**Fig. 1:**
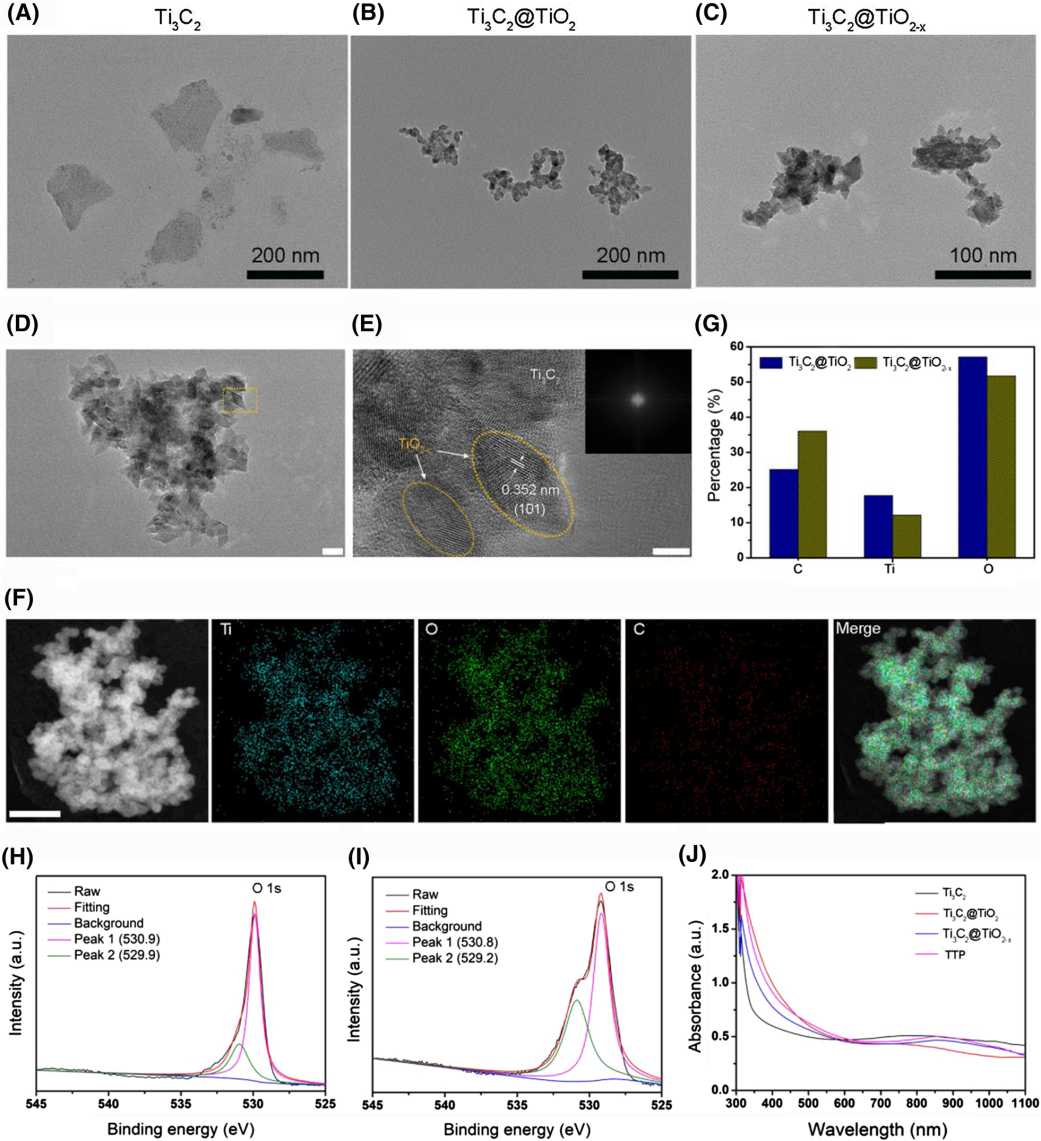
TEM images of **A** Ti_3_C_2_, **B** Ti_3_C_2_@TiO_2_, and **C** Ti_3_C_2_@TiO_2-x_. The scale bars in **A**, **B** and **C** are 200 nm, 200 nm and 100 nm, respectively. **D** and **E** HR-TEM images of the Ti_3_C_2_@TiO_2-x_. The inset in Fig. D is the SAED pattern of TiO_2-x_ taken from the yellow rectangular box in Fig. E. The scale bars in **D** and **E** are 20 and 5 nm, respectively. **F** TEM elemental mapping images of Ti_3_C_2_@TiO_2-x_ hybrid (Ti: blue; O: green; C: red; merged image). Scale bar: 50 nm. **G** Quantitative EDS elemental analysis of Ti_3_C_2_@TiO_2_ and Ti_3_C_2_@TiO_2-x_. **H** O 1 s XPS spectrum of **I** Ti_3_C_2_@TiO_2_ and **G** Ti_3_C_2_@TiO_2-x_. **J** Absorption spectra of Ti_3_C_2_, Ti_3_C_2_@TiO_2_, Ti_3_C_2_@TiO_2-x_, and TTP

The development of TTP nanohybrid was carried out in several steps. In brief, bulk Ti_3_C_2_ powder was first exfoliated via ultrasonication by using TPAOH as an intercalating agent, resulting in the formation of 2D Ti_3_C_2_ nanosheets with an approximate lateral dimension of 150 nm (Fig. 1A). Next, TiO_2_ NPs were grown in situ onto the as-obtained 2D Ti_3_C_2_ nanosheets by hydrothermal method [41]. Compared to the usually prepared TiO_2_ NPs (~ 100 nm) [15, 42, 43], in situ fabricated TiO_2_ NPs were much smaller (~ 10 nm) in diameter as revealed by TEM (Fig. 1B). Considering an inverse relationship between the NPs size and their surface area, the as-obtained TiO_2_ NPs with high surface area could facilitate higher surface adsorption of oxygen molecules and the separation of electron–hole pairs, promoting higher ROS generation [44, 45]. Finally, an engineering of *in-situ* developed Ti_3_C_2_@TiO_2_ was performed through hydrazine hydrate reduction method [46], which induced multiple oxygen defects within TiO_2_ NPs, resulting in the formation of Ti_3_C_2_@TiO_2-x_ nanohybrid. Notably, no apparent aggregation or morphological change was seen after modifications as shown in Fig. 1C, D. The selected area electron diffraction (SAED) pattern of Ti_3_C_2_@TiO_2-x_ (Fig. 1E, inset) indicated the crystalline nature of TiO_2_, whereas the lattice spacings of 0.352 nm were assigned to (101) plane of anatase TiO_2_ [47]. Meanwhile, energy dispersive X-ray spectroscopy (EDS) indicated the presence of titanium, oxygen, and carbon elements in Ti_3_C_2_@TiO_2-x_ (Fig. 1F, G and Additional file 1: Fig. S1A, B). Compared to Ti_3_C_2_ (20 nm), ~ 40–80 nm of height increases were noticed for Ti_3_C_2_@TiO_2_ and Ti_3_C_2_@TiO_2-x_, indicating in situ production and the decoration of TiO_2_ onto Ti_3_C_2_ (Additional file 1: Fig. S2). The X-ray diffraction (XRD) spectrum of TiO_2-x_@Ti_3_C_2_ was well-indexed with TiO_2_ (JCPDS no. 21–1272) and Ti_3_C_2_ (JCPDS no. 32–1383) as shown in Additional file 1: Fig. S3. The elemental composition and oxygen vacancies were further confirmed by XPS spectroscopy. Additional file 1: Fig. S4A–E and Fig. 1H, I verified the presence of titanium (25.92%, 15.7%), oxygen (52.47%, 25.17%) and carbon (21.62%, 44.74%) in Ti_3_C_2_@TiO_2-x_ hybrid, respectively, while O 1 s peak is negatively shifted from 529.9 (Ti_3_C_2_@TiO_2_) to 529.2 eV in Ti_3_C_2_@TiO_2-x_ nanohybrid (Fig. 1H, I), which is attributed to the existence of oxygen vacancies within Ti_3_C_2_@TiO_2-x_, favoring highly efficient ROS production [48, 49]. The quantitative analysis also indicated a prominent decrease in O/Ti ratio from 2 to 1.6 after reduction (Additional file 1: Fig. S4F), implying the creation of oxygen vacancies in Ti_3_C_2_@TiO_2-x_. Such a thorough characterizations proved the successful fabrication of *in-situ* 2D Ti_3_C_2_@TiO_2-x_ nanohybrid with desired oxygen deficiencies to accelerate ROS generation.

Later, Ti_3_C_2_@TiO_2-x_ was surface functionalized with DSPE-PEG, which imparts an aqueous solubility and biocompatibility. Notably, the surface PEGylation did not induce any observable morphological change in the resultant TTP and Ti_3_C_2_@TiO_2_-PEG as shown in Additional file 1: Fig. S5A, B. FT-IR spectra of TTP showed the presence of –CH_2_ and C–O bonds at 2870 and 1100 cm^−1^, respectively, which are assigned to the DSPE-PEG modification (Additional file 1: Fig. S6), while the amount of PEG loaded onto Ti_3_C_2_@TiO_2-x_ hybrid was about 3% as quantitatively determined by TGA (Additional file 1: Fig. S7), indicating the successful surface grafting of Ti_3_C_2_@TiO_2-x_ hybrid by PEG. The hydrodynamic diameter of TTP was about 170 nm as determined by DLS (Additional file 1: Fig. S8), whereas, the surface zeta potential of Ti_3_C_2_@TiO_2_-PEG and TTP was about −7.8 and −11.2 eV, respectively, suggesting limited protein binding and longer blood circulation (Additional file 1: Fig. S9) [50]. Owing to PEG grafting, TTP and Ti_3_C_2_@TiO_2_-PEG exhibited good stability in different physiological solutions such as PBS, Dulbecco’s modified eagle medium (DMEM), and fetal bovine serum (FBS) as shown in Additional file 1: Fig. S10A, B. Meanwhile, no notable change was recorded in the hydrodynamic diameter of TTP and Ti_3_C_2_@TiO_2_-PEG under different physiological solutions for 3 days, verifying the excellent colloidal stability of TTP (Additional file 1: Fig. S10C, D). UV–vis/NIR absorption spectroscopy indicated the broadband absorption of TTP in NIR-II region without any particular absorption peak (Fig. 1J), which is solely ascribed to the presence of 2D Ti_3_C_2_ sheets, suggesting the potential of as-designed TTP hybrid for dual-modal PT/PA imaging and PTT at second biowindow.

### Evaluation of photothermal and sonodynamic capacity

**Fig. 2:**
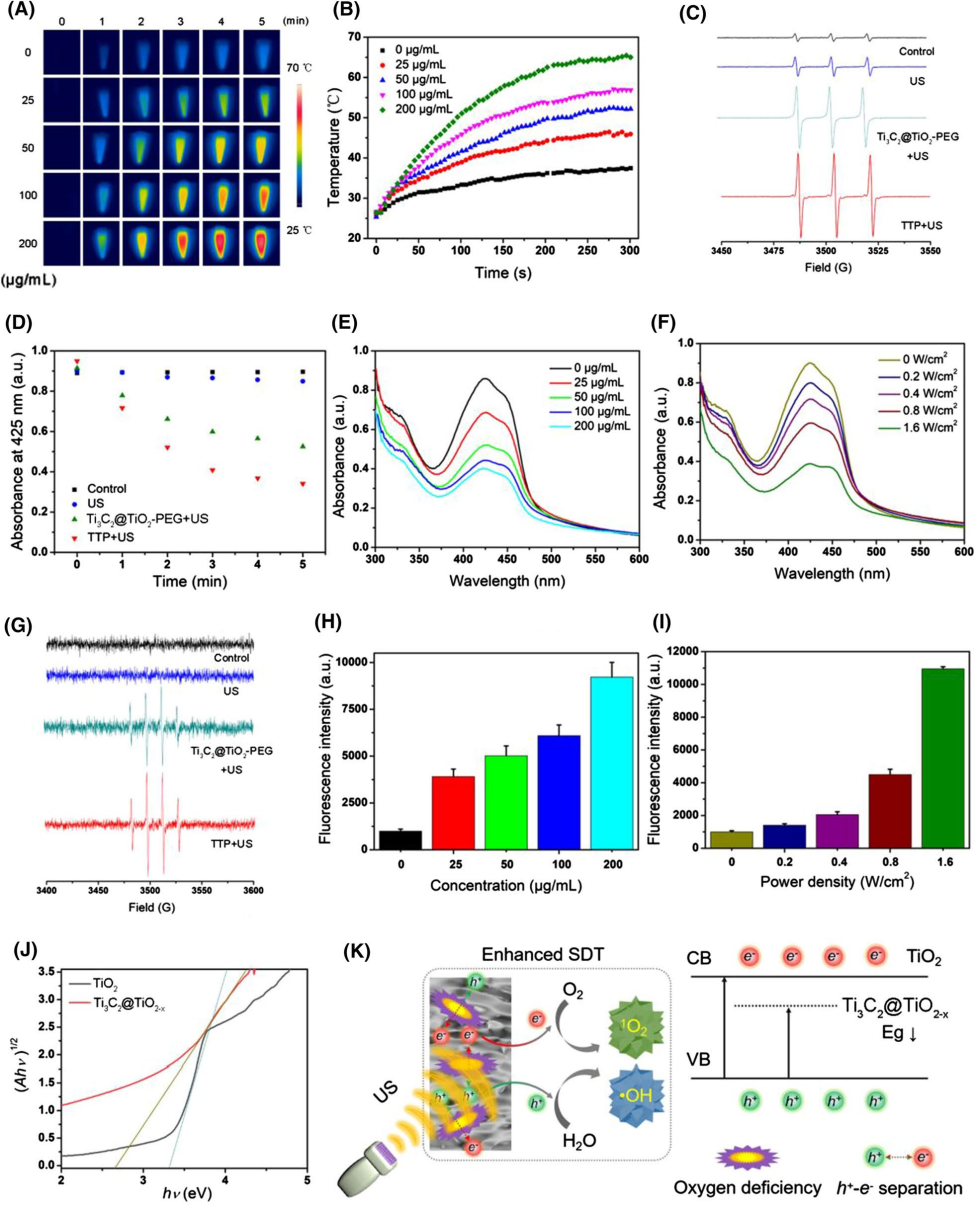
**A** The real-time thermal images and **B** the corresponding heating curves of the varying concentrations of TTP aqueous solutions. **C** ESR spectra of ^1^O_2_/TEMP adduct by incubating Ti_3_C_2_@TiO_2_-PEG or TTP with TEMP (20 mM). **D** The absorbance profile of DPBF at 425 nm under different conditions and US stimulation (1 W/cm^2^, 1 MHz) for 5 min. **E** The absorbance spectra of DPBF treated with different concentrations (0–200 μg/mL) of TTP under US irradiation (1 W/cm^2^, 1 MHz, 5 min). **F** The absorbance spectra of DPBF treated with 50 μg/mL of TTP under US irradiations with different power density (0–1.6 W/cm^2^, 1 MHz, 5 min). **G** ESR spectra of ·OH/DMPO adduct by incubating Ti_3_C_2_@TiO_2_-PEG or TTP with DMPO (10 mM). **H** The fluorescence intensity of NaOH solution (2 mM) containing TPA (0.5 mM) and treated with different concentrations of TTP, followed by US irradiation (1 W/cm^2^, 1 MHz, 3 min). **I** The fluorescence intensity of NaOH solution containing TPA (0.5 mM) and TTP (50 μg/mL) irradiated with US of different power density (0–1.6 W/cm^2^, 1 MHz, 3 min). **J** Optical band gaps of TTP and TiO_2_. **K** Schematic illustration of the US-induced ROS generation by TTP hybrid, and the proposed mechanism of accelerated sonodynamic performance of TTP by enhanced separation of charge carriers through oxygen defects

Considering the good NIR-II absorption, the photothermal capacity of TTP was investigated. Figure 2A, B displayed significant temperature enhancement in TTP solution under 1064 nm laser irradiation (0.8 W/cm^2^) for 5 min, which is directly proportional to the irradiation time and TTP concentration, respectively. Furthermore, the calculated photothermal conversion efficiency (PCE, *η*) of TTP was about 35.8% (Additional file 1: Fig. S11A, B) [51], which is superior or even comparable to many previously reported photothermal nanoagents [52–55]. Notably, under 5 repetitive laser on/off cycles, no apparent temperature decrease was recorded as shown in Additional file 1: Fig. S12A. Moreover, the absorption spectrum and TEM morphological characterization did not show any particular change in TTP even after 1064 nm laser irradiation for 20 min, suggesting the excellent photothermal stability of TTP (Additional file 1: Fig. S12B, C). Inorganic TiO_2_ nanoparticles have been widely reported as sonosensitizers, capable of ROS production such as singlet oxygen (^1^O_2_) and hydroxyl radicals (·OH), etc. under US irradiation [17, 18, 42, 56, 57]. Therefore, electron spin resonance (ESR) spectroscopy was employed to determine the ROS generation by TTP under US activation. Figure 2C presented the characteristic (1:1:1) peaks of ^1^O_2_ after US stimulation of both Ti_3_C_2_@TiO_2_-PEG and TTP group, respectively. The ^1^O_2_ generation ability of TTP was further evaluated by using 1, 3-diphenylisobenzofuran (DPBF) probe. Figure 2D and Additional file 1: Fig. S13 showed time-dependent gradual decrease in DPBF absorbance at 410 nm by both Ti_3_C_2_@TiO_2_-PEG and TTP group, while the control group did not reduce the DPBF absorption at all. It is worth mentioning that TTP showed stronger ^1^O_2_ production capacity than Ti_3_C_2_@TiO_2_-PEG, which is possibly ascribed to the presence of several oxygen vacancies within TiO_2-x._ Notably, these oxygen vacancies played a vital role as they promoted effective separation of charge carriers (electron–hole pairs), and thus facilitate an enhanced interaction between charge carriers and surface adsorbed oxygen molecules, leading to enhanced ROS generation. Meanwhile, the concentration and US power-dependent ^1^O_2_ production was also recorded by TTP as shown in Fig. 2E, F and Additional file 1: Fig. S14A, B. Besides ^1^O_2_, the characteristic (1:2:2:1) peaks of 5,5-Dimethyl-1-pyrroline N-oxide (DMPO) and ·OH adducts were appeared both in TiO_2_@Ti_3_C_2_-PEG and TTP groups, indicating the generation of ·OH ions (Fig. 2G). Similarly, a ·OH indicator TPA, which emits fluorescence after reacting with ·OH under UV light, was also used to detect the ability of TTP to produce ·OH under US stimulation. As shown in Additional file 1: Fig. S15, a strong fluorescence signal is generated at 422 nm after Ti_3_C_2_@TiO_2_-PEG and TTP activation by US. However, the control group and US irradiation alone showed much weaker fluorescence signal. The quantitative analysis suggested that TTP has a stronger ·OH production ability than Ti_3_C_2_@TiO_2_-PEG. Similar to ^1^O_2_, the production of ·OH is also dependent on TTP concentration and US power density (Fig. 2H, I). These results verified that TTP holds excellent ^1^O_2_ and ·OH production capacity under ultrasonic stimulation. For better apprehension of the sonodynamic performance of Ti_3_C_2_@TiO_2-x_, the band gap calculation was made by Kubelka–Munk function, which was about 3.32 and 2.65 eV for TiO_2_ and Ti_3_C_2_@TiO_2-x_, respectively (Fig. 2J). Such a narrow band gap of Ti_3_C_2_@TiO_2-x_ than TiO_2_ NPs could be ascribed to the overlapping oxygen deficiency induced defect states with the band edge of the semiconductor. Thereby, Ti_3_C_2_@TiO_2-x2_ served as a charge trap, and remarkably restricts the charge recombination (electron–hole pairs) under US excitation, promoting an enhanced sonodynamic ROS generation during SDT (Fig. 2K).

### In vitro combined PTT/SDT

**Fig. 3:**
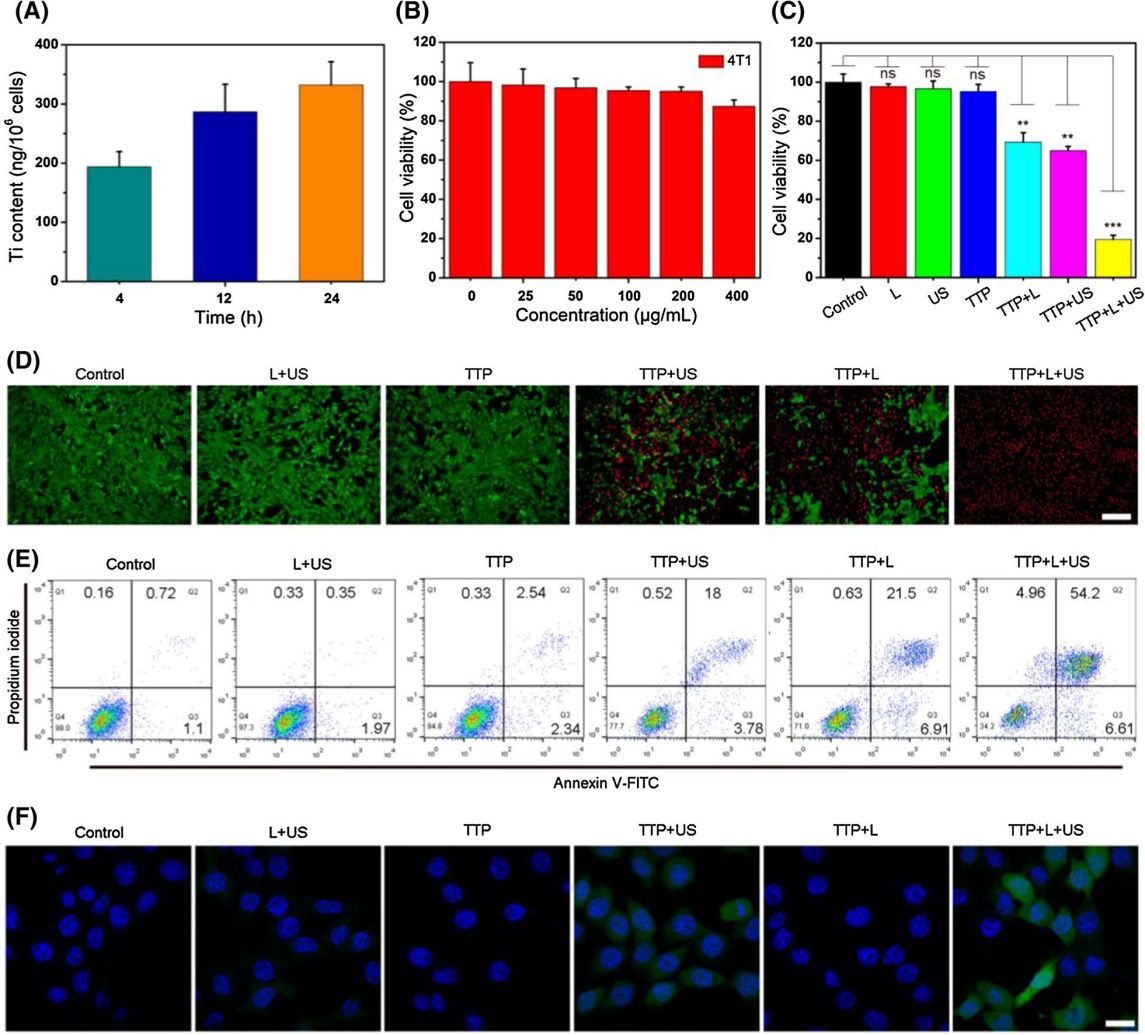
**A** Estimation of Ti content in 4T1 cells after treated with TTP at indicated time points as determined by ICP-MS. **B** Relative viabilities of 4T1 cells treated with various concentrations of TTP nanohybrids for 24 h in dark. **C** Cellular viability of 4T1 cells incubated with TTP (100 μg/mL) under different conditions like: (I) Control, (II) Laser, (III) US, (IV) TTP, (V) TTP + US, (VI) TTP + Laser, and (VII) TTP + Laser + US. Statistical significance compared with the control group is shown (**p < 0.01 and ***p < 0.001). **D** Confocal laser scanning microscopic (CLSM) images of 4T1 cells stained by Calcein-AM and PI after different treatments as mentioned. The scale bar is 50 μm. **E** Flow cytometric analysis of 4T1 cells after different indicated treatments. **F** CLSM images of in vitro ROS generation in 4T1 cells as stained by DCF (green) after different treatments, while the cell nucleus was stained by Hoechst 33,342 dye (blue). The scale bar is 10 μm

The in vitro cellular uptake of TTP was determined by ICP-MS. The cellular uptake mechanism of TTP was investigated by using various inhibitors. As presented in Additional file 1: Fig. S16, the cellular uptake of TTP was significantly reduced when incubated with chlorpromazine or at 4 ℃, suggesting that TTP entered into cells through an energy-dependent and clathrin-mediated endocytosis pathway. As shown in Fig. 3A, the Ti content in 4T1 cells increases gradually with an increase in incubation time, indicating an efficient cellular internalization of TTP. While, MTT assay revealed no potential cytotoxicity of internalized TTP on 4T1 and human embryonic kidney (HEK293T) cells in dark, suggesting good biocompatibility (Fig. 3B and Additional file 1: Fig. S17). However, TTP substantially reduced the cellular viability of 4T1 cells under either 1064 nm laser (PTT) or US (SDT) activation, respectively. The cellular viability was reduced up to 45% and 39% by individual PTT and SDT treatment alone (Fig. 3C), whereas, the combined PTT/SDT triggered significant cellular killing, resulting in a dramatic decrease (16.5%) in cells viability, suggesting superior therapeutic killing performance of combined PTT/SDT treatment than either PTT or SDT alone. MTT findings were also further endorsed by live and dead assay (Fig. 3D) and Annexin V-FITC and PI staining (Fig. 3E) as much higher proportion of dead cells (apoptosis and necrosis) were observed after dual laser/US treatment (Additional file 1: Fig. S18), verifying an extensive in vitro cellular killing by synergistic PTT/SDT.

Considering that ^1^O_2_ and ·OH can be produced under ultrasonic stimulation in an aqueous solution. The ROS probe 2,7-dichlorofluorescein diacetate (DCFH-DA) was utilized to assess ROS production in vitro in 4T1 cells. Negligible green fluorescence was noticed in cells after treated with US, laser, or TTP alone, while an obvious green fluorescence was seen after simultaneous treatment with TTP and US (Fig. 3F), implying significant ROS production.

### In vivo biocompatibility

**Fig. 4:**
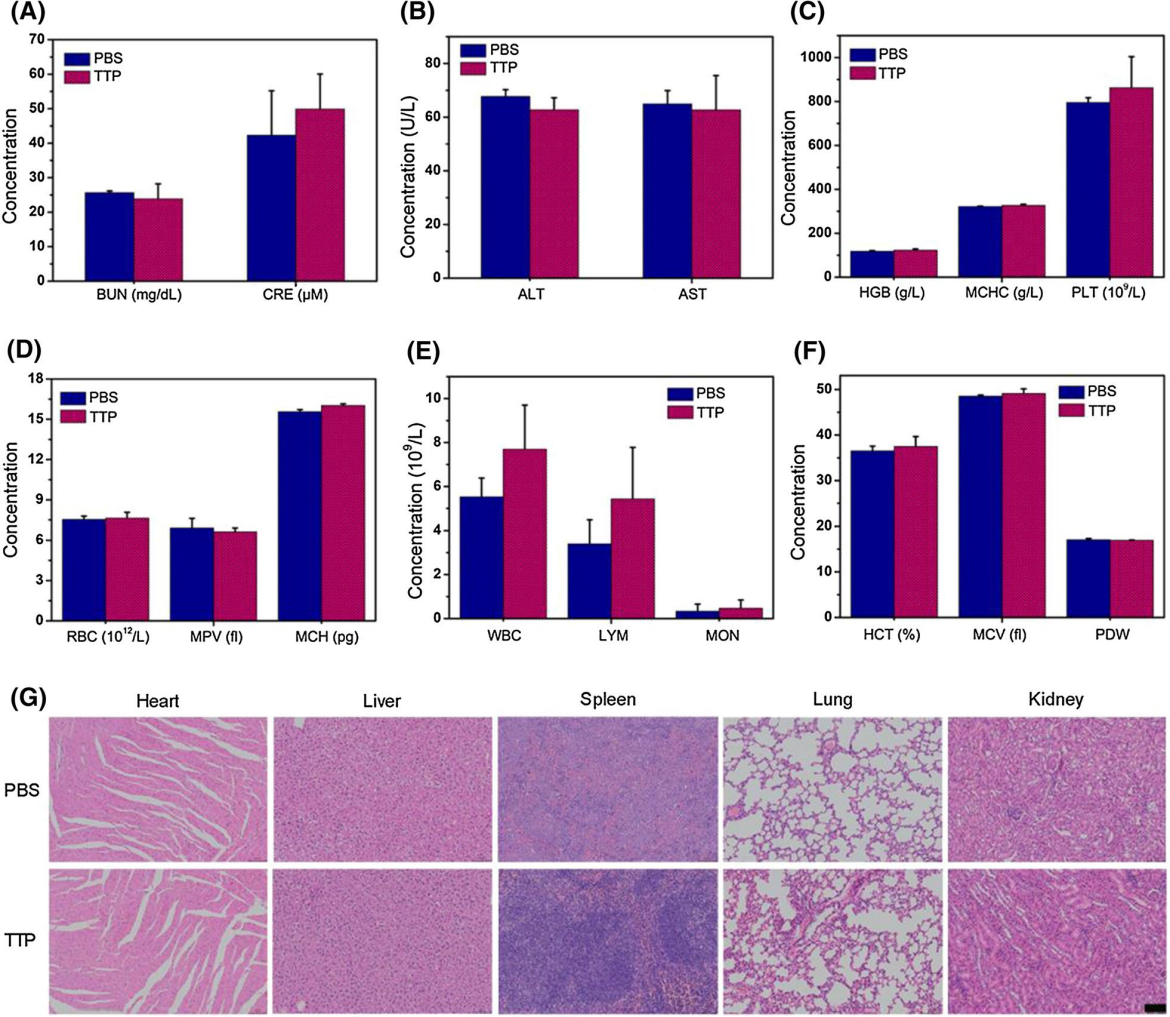
**A** Different levels of kidney and liver function indicators **B** in serum as mentioned after different treatments. Blood panel analysis: **C** hemoglobin (HGB), mean corpuscular hemoglobin concentration (MCHC), and platelets (PLT); **D** red blood cells (RBC), mean platelet volume (MPV), mean corpuscular hemoglobin (MCH); **E** white blood cells (WBC), lymphocyte (LYM), monocyte (MON); **F** hematocrit (HCT), mean corpuscular volume (MCV), and platelet distribution width (PDW) of mice treated with PBS or TTP (4 mg/mL, 200 μL). **G** H&E staining of major organs after different treatments. The scale bar is 100 μm

Biosafety is an important prerequisite for the development of nanodrugs in biomedical field. Hence, the in vivo biocompatibility of TTP was evaluated by hemolysis assay, hematology analysis, and H&E staining of the major organs (heart, liver, spleen, lungs, and kidneys). Additional file 1: Fig. S19 showed no adverse effects of TTP on red blood cells, while all the hematological parameters, including liver (aspartate transaminase, AST, alanine aminotransferase, ALT), kidney function (blood urea nitrogen, BUN, creatinine, CRE), and other blood biochemical indicators as shown in Fig. 4A–F, were also in normal range like PBS-treated control mice. In addition, H&E analysis did not display any apparent inflammation in major organs (Fig. 4G). Moreover, like PBS-treated mice, no particular change was recorded in the body weight of TTP-treated mice even after one month (Additional file 1: Fig. S20). Collectively, these results verified the good biocompatibility and biosafety of TTP in vivo.

### In vivo biodistribution and duplex PA/PT imaging

**Fig. 5:**
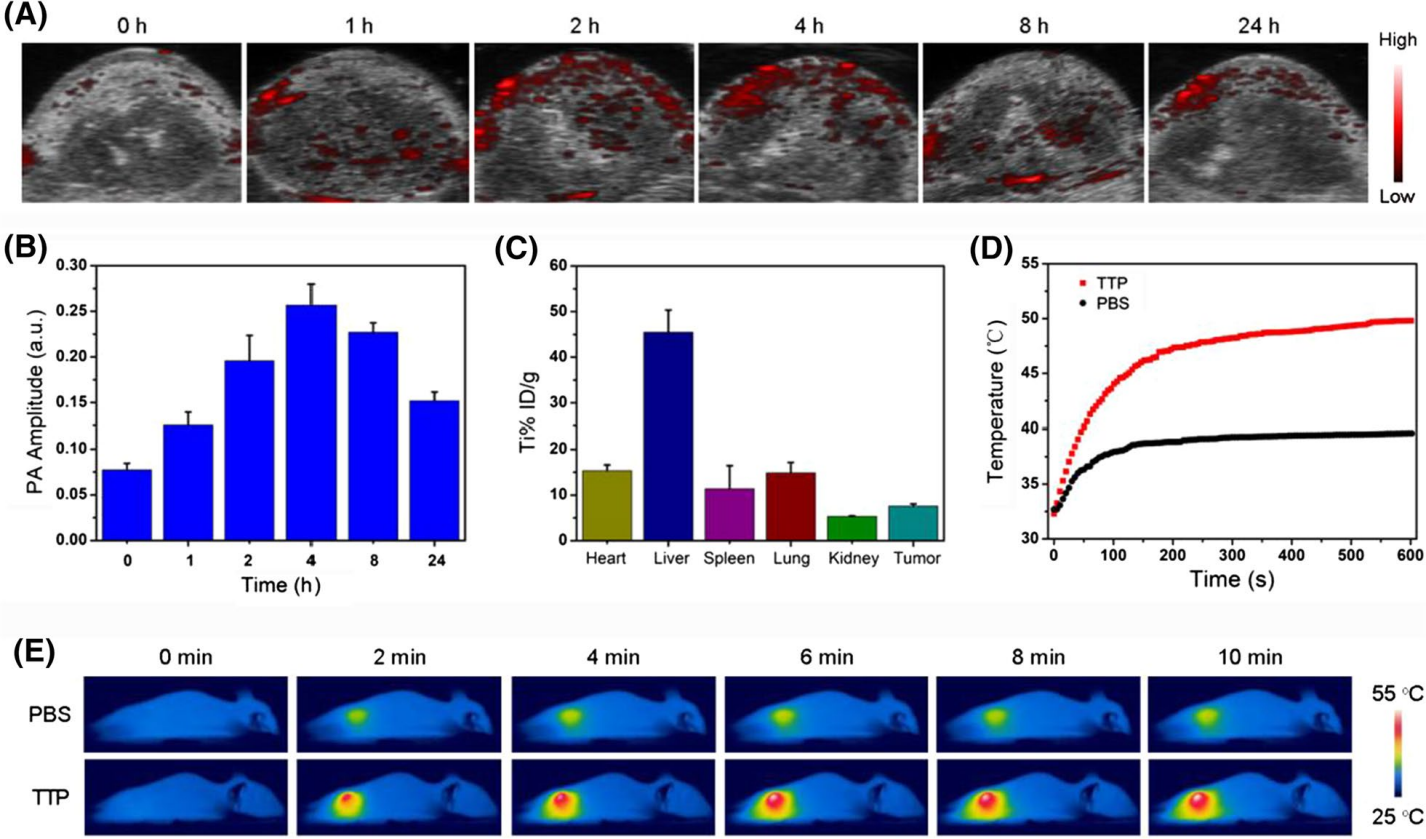
**A** PA images and **B** the corresponding PA signal intensity of tumor at mentioned time points after *i.v.* injection of TTP (2 mg/mL, 200 μL). **C** The estimation of Ti content in vivo by ICP-MS. **D** The real-time temperature increase and **E** the thermal imaging of mice at tumor tissues after treated with/without TTP under 1064 nm laser irradiation (0.8 W/cm^2^, 10 min)

Prior to the determination of in vivo therapeutic potential of TPP, we assessed the in vivo biodistribution of TTP by PA imaging and ICP-MS, respectively. As expected, the PA signal intensity was positively correlated with the concentration of TTP (Additional file 1: Fig. S21). As can be seen in Fig. 5A, B, compared to pre-injection, the PA signal intensity is significantly enhanced in tumor tissues after post-injection, indicating an effective tumor accumulation of TTP. Importantly, the highest PA signal was found at 4 h post-injection, which provides an effective guidance and optimal treatment time for tumor PTT in vivo. Next, the Ti content in major organs of mice was detected by ICP-MS. Figure 5C displayed prominent accumulation of TTP in reticuloendothelial system *e.g.*, liver and spleen, whereas, 5% ID/g of TTP was found in tumor tissues, which is advantageous for combined tumor PTT/SDT in vivo. Besides PA imaging and ICP-MS, in vivo PT imaging was also performed to determine the temperature enhancement at tumor site by intravenously injected TTP under 1064 nm laser excitation for 10 min. The temperature of TTP-treated mice was abruptly increased from 28 to 52 ℃, while a slight temperature change was noticed in PBS group (Fig. 5D, E). These results demonstrated the good photothermal performance of TTP in vivo.

### In vivo synergistic PTT/enhanced SDT

**Fig. 6:**
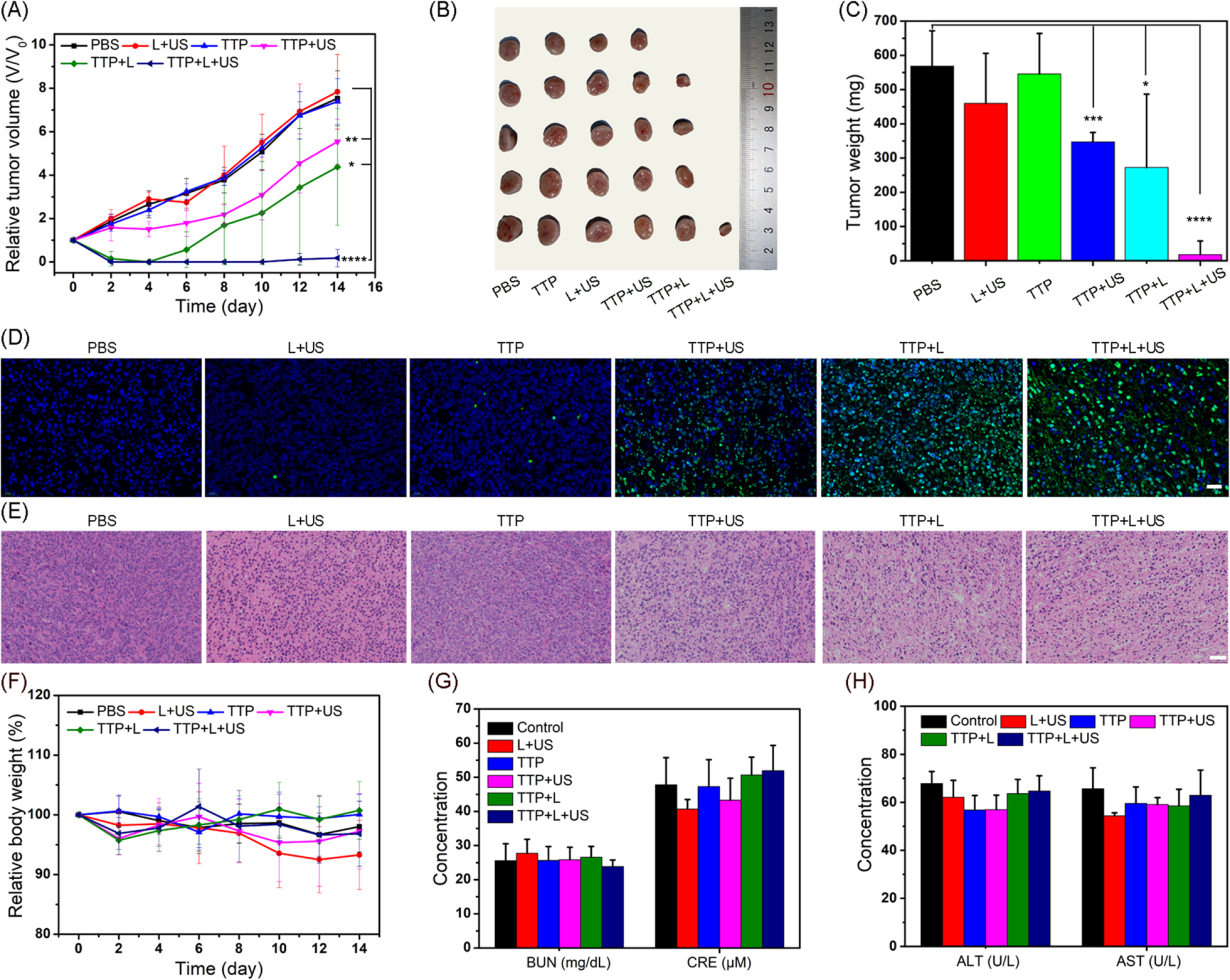
**A** Relative change in tumor volume after different treatments. **B** The digital images and **C** the weight of tumors collected from different indicated groups at 14th day. **D** TUNEL assay and **E** H&E-stained images of tumor tissues after different treatments at day 14th. The scale bars are 50 and 100 μm in **D** and **E**, respectively. **F** Relative change in the body weight of mice during different treatments. Levels of different blood biochemical indicators **G** BUN and CRE, **H** ALT and AST in mice serum after different treatments for 14 days. Statistical significance compared with the PBS group is shown (*p < 0.05; **p < 0.01, ***p < 0.001 and ****p < 0.0001)

Finally, the therapeutic efficacy of synergistic PTT/enhanced SDT triggered by TTP was investigated. 4T1 tumor-bearing mice with a mean tumor volume of about 80 mm^3^ were randomly assigned to seven groups, and received an *i.v.* injection of either TTP (20 mg/kg) or PBS, respectively. The tumor volume of each mice undergo different treatment conditions, was recorded every two days, while after 14 days, all the tumors were dissected, weighed, and photographed. As Additional file 1: d the tumor growth, whereas the combined PTT/SDT triggered complete tumor eradication. The in vivo therapeutic effects were further evaluated by TUNEL assay, which showed the highest green fluorescence intensity in the combined TPP + Laser + US treatment group (Fig. 6D), indicating substantial cellular apoptosis. Similarly, the H&E staining of tumor tissues verified the superior therapeutic effects as noticeable nuclear shrinkage and reduced number of cancer cells (Fig. 6E) were found in combined treatment than individual PTT or SDT alone. These results are in accordance with the in vitro findings. Remarkably, the body weight of the mice in each group was remained stable after different treatments (Fig. 6F). In addition, no significant lesions or damages were found in the major organs as confirmed by H&E staining (Additional file 1: Fig. S23) and blood biochemistry analysis (Fig. 6G, H), implying the negligible systemic toxicity of TTP in vivo.

## Conclusion

In summary, TTP was prepared by the *in-situ* fabrication of ~ 10 nm TiO_2-x_ NPs with multiple oxygen defects onto the Ti_3_C_2_ nanosheets, followed by PEG grafting, offering synergistic PTT/enhanced SDT under PA/PT bimodal imaging guidance in second biowindow. TTP possessed good colloidal stability, excellent photothermal conversion efficiency, and absorption in NIR-II region. Importantly, TTP with higher surface area and several oxygen-defects, facilitated higher surface adsorption of O_2_ molecules as well as an efficient separation of charge carriers under US activation, promoting an enhanced interaction of charge carriers with surface adsorbed O_2_ to trigger higher sonodynamic ROS generation than commonly reported TiO_2_ NPs. Owing to good photothermal and enhanced US-stimulated ROS production capacity, TTP showed high therapeutic efficiency in vitro with appreciable biocompatibility, and demonstrated complete tumor elimination in 4T1 tumor bearing mice in vivo*,* because of synergistic photothermal enhanced SDT, which is far greater than single treatment modality. Our work demonstrated the design engineering of hybrid nanosystems for enhanced theranostics of deeply localized tumors in clinic.

## Supplementary Information

**Additional file 1.** Additional information includes part of material and methods, additional figures and tables.

## Data Availability

All data used to generate these results is available in the main text and supporting information.

## Abbreviations

NIR-I: Near-infrared-I
ROS: Reactive oxygen species
PTT: Photothermal therapy
US: Ultrasound
SDT: Sonodynamic therapy
TiO_2_: Titanium dioxide
Ti_3_C_2_: Titanium carbide
PA: Photoacoustic;
PEG: Polyethylene glycol
TPAOH: Tetrapropylammonium hydroxide
DSPE-PEG: 1,2-Distearoyl-sn-glycero-3-phosphoethanolamine-N-[methoxy(polyethylene glycol)
TPA: Terephthalic acid
MTT: 3-(4,5)-Dimethylthiahiazo (-z-y1)-3,5-di-phenytetrazoliumromide
PI: Propidium iodide
TEM: Transmission electron microscope
XPS: X-ray photoelectron spectrometer
FT-IR: Fourier transform infrared
TGA: Thermo-gravimetric analysis
DLS: Dynamic light scattering
ICP-MS: Inductively coupled plasma mass spectrometry
TTP: Ti_3_C_2_@TiO_2-x_-PEG
PBS: Phosphate buffered saline
H&E: Hematoxylin and eosin
TUNEL: Terminal-deoxynucleoitidyl transferase mediated nick end labeling
SAED: Selected area electron diffraction
EDS: Energy dispersive X-ray spectroscopy
XRD: X-ray diffraction
DMEM: Dulbecco’s modified eagle medium
FBS: Fetal bovine serum
^1^O_2_: Singlet oxygen
·OH: Hydroxyl radicals
ESR: Electron spin resonance
DPBF: 1, 3-Diphenylisobenzofuran
DMPO: 5,5-Dimethyl-1-pyrroline N-oxide
HEK 293 T: Human embryonic kidney 293 T
DCFH-DA: 2,7-Dichlorofluorescein diacetate
AST: Aspartate transaminase
ALT: Alanine aminotransferase
BUN: Blood urea nitrogen
CRE: Creatinine
HGB: Hemoglobin
MCHC: Mean corpuscular hemoglobin concentration
PLT: Platelets
RBC: Red blood cells
MPV: Mean platelet volume
MCH: Mean corpuscular hemoglobin
WBC: White blood cells
LYM: Lymphocyte
MON: Monocyte
HCT: Hematocrit
MCV: Mean corpuscular volume
PDW: Platelet distribution width
O_2_: Oxygen
