# Supplementary material for: In-situ TiO2-x decoration of titanium carbide MXene for photo/sono-responsive antitumor theranostics
Source: J Nanobiotechnology. 2022 Jan 28;20:53. doi: 10.1186/s12951-022-01253-8 (PMC8796495; doi:10.1186/s12951-022-01253-8)
Supplement: Supplementary file 1 — Additional file 1. Additional information includes part of material and methods, additional figures and tables. [file 12951_2022_1253_MOESM1_ESM.docx]

Supporting Information

*In-Situ* TiO_2-x_ Decoration of Titanium Carbide MXene for Photo/Sono-Responsive Antitumor Theranostics

Dong-Yang Zhang^1,2,†^, Hengke Liu^2,†^, Muhammad Rizwan Younis^2,†^, Shan Lei^2^, Yunzhi Chen^1^, Peng Huang^2*^, and Jing Lin^1,2*^

^1^Department of Hepatobiliary Surgery, The First Affiliated Hospital of Wenzhou Medical University, Wenzhou, Zhejiang 325000, China.

^2^Marshall Laboratory of Biomedical Engineering, International Cancer Center, Laboratory of Evolutionary Theranostics (LET), School of Biomedical Engineering, Shenzhen University Health Science Center, Shenzhen 518060, China

Corresponding authors Email: [jingl@szu.edu.cn;](mailto:jingl@szu.edu.cn;) [peng.huang@szu.edu.cn](mailto:peng.huang@szu.edu.cn).

^†^These authors contributed equally: Dong-Yang Zhang, Hengke Liu and Muhammad Rizwan Younis.

Material and methods

**Materials**

Ti_3_AlC_2_ powder (200-meshes) was purchased from Beijing Huawei Ruike Chemical Co., Ltd (China). Hydrofluoric acid (40%, purity >98%) was obtained from Sigma Aldrich (USA). Ammonium fluoride, TPAOH, and hydrazine hydrate were purchased from Macklin (China). DSPE-PEG (MW=2000) was purchased from Shanghai Pengsheng Biotechnology Co., Ltd (China). TPA and MTT were obtained from J&K Reagent (China). Calcein AM, PI, and Annexin V-FITC were bought from Beyotime Biotechnology (China).

**Instrumentation**

Transmission electron microscope (JEM-2100F, JEOL, Japan) and (HT7700, HITACHI, Japan) were used to image the nanomaterials. The crystal structure of nanomaterials was analyzed by an X-ray diffractometer (PANalytical Empyrean, Netherlands). The XPS spectra were detected by an X-ray photoelectron spectrometer (K-Alpha+, Thermo Fisher Scientific, UK). FT-IR spectra were obtained by using an infrared spectrophotometer (L16000300 Spectrum TWO LITA, Llantrisant, UK). TGA was carried out by a [thermal](E:/%E6%99%AE%E9%80%9A%E8%BD%AF%E4%BB%B6/%E6%9C%89%E9%81%93%E8%AF%8D%E5%85%B8/Dict/8.9.6.0/resultui/html/index.html#/javascript:;) [gravimetric](E:/%E6%99%AE%E9%80%9A%E8%BD%AF%E4%BB%B6/%E6%9C%89%E9%81%93%E8%AF%8D%E5%85%B8/Dict/8.9.6.0/resultui/html/index.html#/javascript:;) [analyzer](E:/%E6%99%AE%E9%80%9A%E8%BD%AF%E4%BB%B6/%E6%9C%89%E9%81%93%E8%AF%8D%E5%85%B8/Dict/8.9.6.0/resultui/html/index.html#/javascript:;) (Pyris 1, Perkin Elmer, UK). DLS particle size analyzer (Malvern 2000, USA) was utilized to determine the size of the nanoparticles. An absorption spectrophotometer (Cary 60, Agilent Technologies, USA) was used to measure the absorption spectra of nanomaterials. The concentration of Ti was measured by using an ICP-MS (PerkinElmer, NexIon300X, USA).

**Photothermal performance of TTP nanohybrids**

The aqueous solutions of TTP nanohybrids (0-200 μg/mL) were placed in centrifuge tubes and irradiated by a 1064 nm laser at 0.8 W/cm^2^ for 5 min. The temperature changes in the solutions were recorded by a thermal imager. Water was used as a control. The photothermal conversion efficiency of nanohybrids was calculated according to the reported literature [1]. The photothermal stability of nanohybrids was studied by cyclic irradiation of a 1064 nm laser at 0.8 W/cm^2^.

**ROS generation from TTP nanohybrids by US-triggered**
The generation of different types of reactive oxygen species (ROS) was identified by electron spin resonance spectroscopy (ESR) experiments using the singlet oxygen probe TEMP and hydroxyl radical probe DMPO under US (1 W/cm^2^, 1 MHz) irradiation. 50 µg/mL of TTP nanohybrid was added to TEMP (50 mM) or DMPO (20 mM) with/without US (1 W/cm^2^, 1 MHz) irradiation for 3 min. The ESR signals were determined by an ESR spectrometer.

The generation of ^1^O_2_ was further measured using DPBF as the ROS indicator. The absorbance values at 425 nm of solutions containing TTP nanohybrid (50 µg/mL) and DPBF (60 μM) with/without US irradiation for 5 min, were recorded. Similarly, the ·OH generation from TTP nanohybrids with/without US (1 W/cm^2^, 1 MHz) irradiation was also detected with TPA by measuring the fluorescence intensity of NaOH solutions (2 mM) containing nanohybrids (100 µg/mL) and TPA (0.5 mM). The excitation wavelength is 312 nm and the emission wavelength is 422 nm.

**Cellular uptake**

4T1 cells were seeded into 10 cm dishes and incubated for overnight. Then, the cells were treated with TTP nanohybrid (100 µg/mL). After 4 or 12 h incubation, the cells were collected after washing with PBS. Next, the cells were digested with aqua regia and measured by ICP-MS. The cells were treated with PBS or various inhibitors, including amiloride (a macropinocytosis inhibitor, 100 μg/mL), chlorpromazine (a clathrin-mediated uptake inhibitor, 10 μg/mL) and nystatin (caveolin-mediated uptake inhibitor, 15 μg/mL) in a serum-free DMEM medium at 37 °C for 0.5 h, and PBS was used at 4 °C. Then, the cells were treated with TTP (100 μg/mL) for 4 h. Subsequently, the cells were washed several times with PBS, digested with aqua regia, and the Ti content was determined by ICP-MS.

***In vitro* cytotoxicity assay**
Using 96-well plates, the 4T1 cells (10^4^ cells/well) were seeded and cultured for overnight. Then, various concentrations of TTP nanohybrid (0-400 μg/mL) were added and incubated for next 20 h. Then, the cells were treated with MTT for 4 h. After replaced the medium by DMSO, the absorbance values of each well at 490 nm were recorded. For the PTT or SDT treatment, the cells were treated with laser irradiation (1064 nm, 0.8 W/cm^2^) for 5 min and US (1 W/cm^2^, 1 MHz) for 5 min, respectively. For combined PTT/SDT, the cells were sequentially irradiated with laser irradiation (1064 nm, 0.8 W/cm^2^) for 5 min and then with US (1 W/cm^2^, 1 MHz) for 5 min. The cell viability was calculated based on the previous literature [1].

The live and dead cells were stained by using Calcein AM and PI, respectively. The 4T1 cells were treated with/wihtout 100 μg/mL TTP for 4 h. Then, the cells were irradiated by a NIR-II laser (1064 nm, 0.8 W/cm^2^, 5 min) and/or US (1 W/cm^2^, 1 MHz, 5 min). After incubation with the above-mentioned two dyes, the cells were imaged by a confocal laser scanning microscopy (CLSM, Zeiss, LSM880, Germany).

The 4T1 cells (10^5^ cells/well) were seeded into 6-well plates. After incubated with TTP (100 μg/mL) for 4 h, the cells were irradiated by a NIR-II laser (1064 nm, 0.8 W/cm^2^, 5 min) and/or US (1 W/cm^2^, 1 MHz, 5 min). After incubation for another 12 h, the cells were stained by Annexin V-FITC/PI and detected by a flow cytometry (FACSCalibur, BD company, USA).

**Detection of intracellular ROS**

4T1 cells were seeded in 35 mm dishes and incubated for overnight. The cells were further incubated with TTP nanohybrids (100 µg/mL) for 12 h, and treated with/without US for 5 min (1 W/cm^2^, 1 MHz). After washed with PBS, the cells were stained by DCF (5 μM, Sigma Aldrich, USA) for 10 min. Then, the cells were imaged by a confocal microscopy. The excitation is 488 nm and the emission is 530 ± 30 nm.

**Statistical analysis**

All data were expressed as a mean ± standard deviation. The difference between the biological tests was calculated by T test (****p* < 0.001, ***p* < 0.01, **p* < 0.05, ns: non-significant).


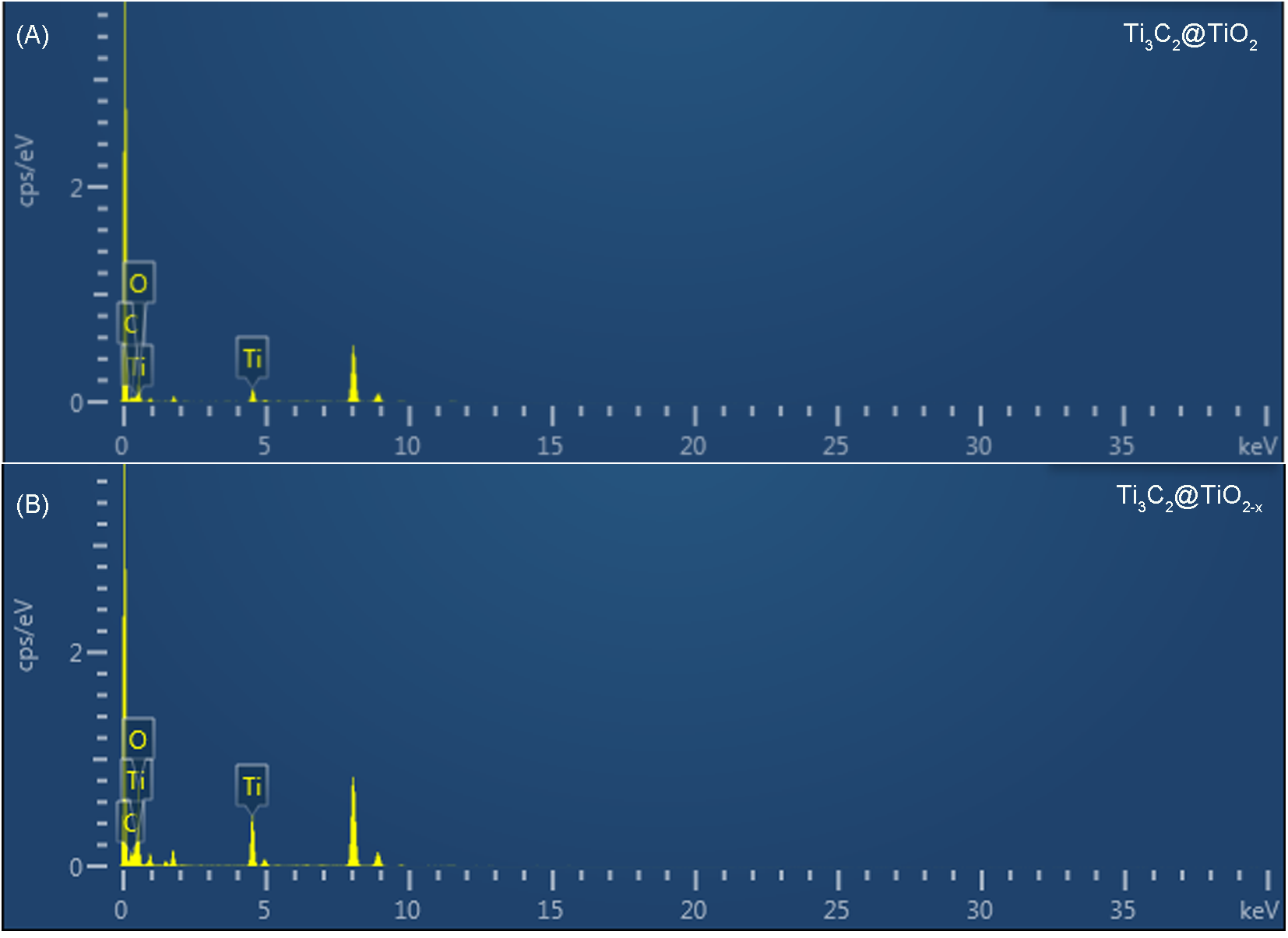


Figure S1. Energy dispersive X-ray spectra of Ti_3_C_2_@TiO_2_ and Ti_3_C_2_@TiO_2-x_, respectively.


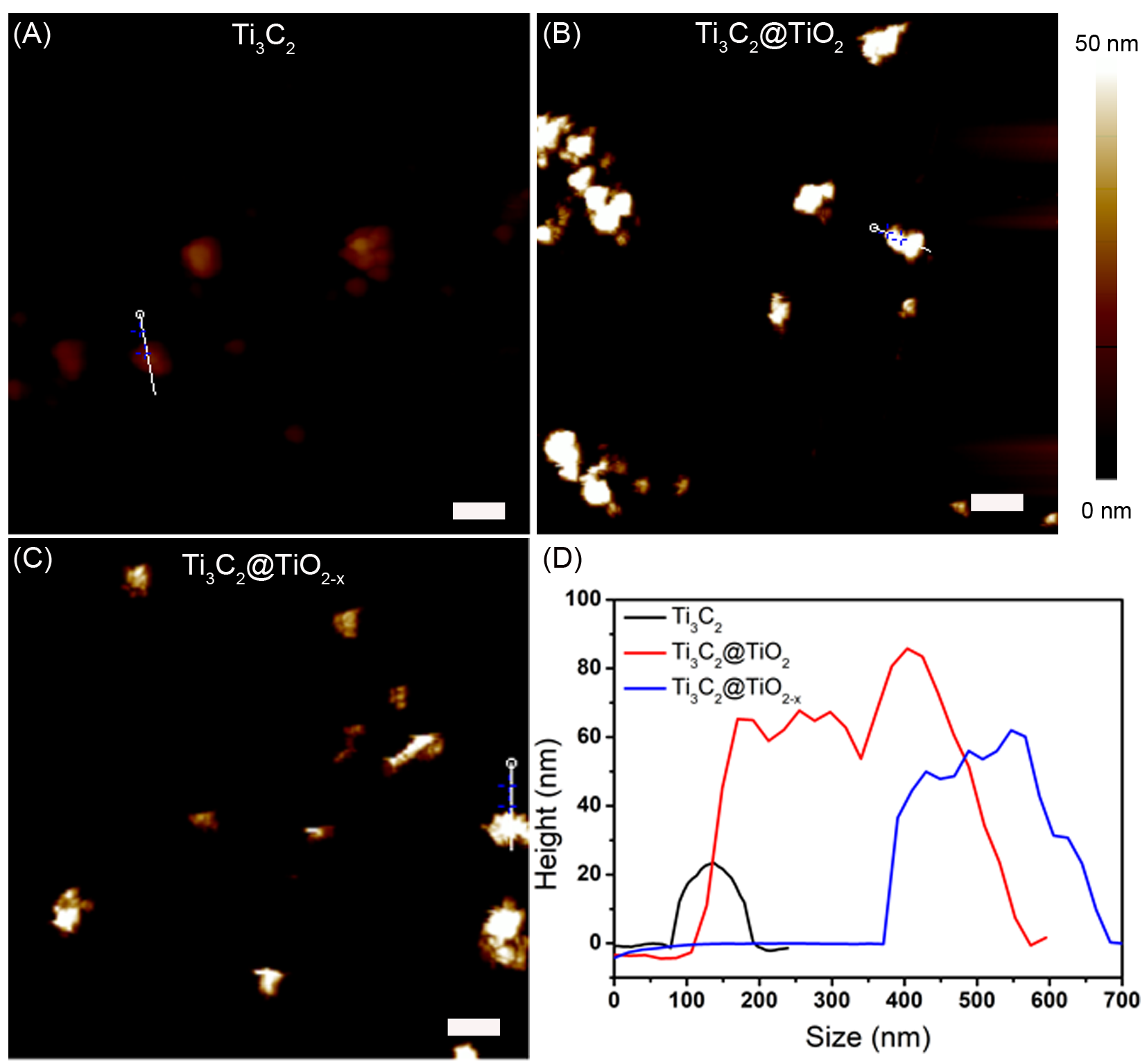


Figure S2. The atomic force microscopy images of (A) Ti_3_C_2_, (B) Ti_3_C_2_@TiO_2_ and (C) Ti_3_C_2_@TiO_2-x_ and (D) the corresponding height profile. The scale bars are 150 nm (A) and 500 nm (B-C), respectively.


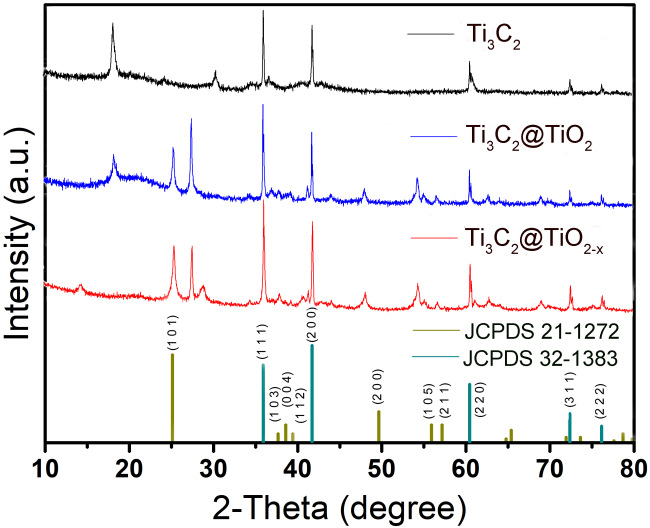


Figure S3. XRD pattern of Ti_3_C_2_, Ti_3_C_2_@TiO_2_, Ti_3_C_2_@TiO_2-x_, and the peaks of standard cards.


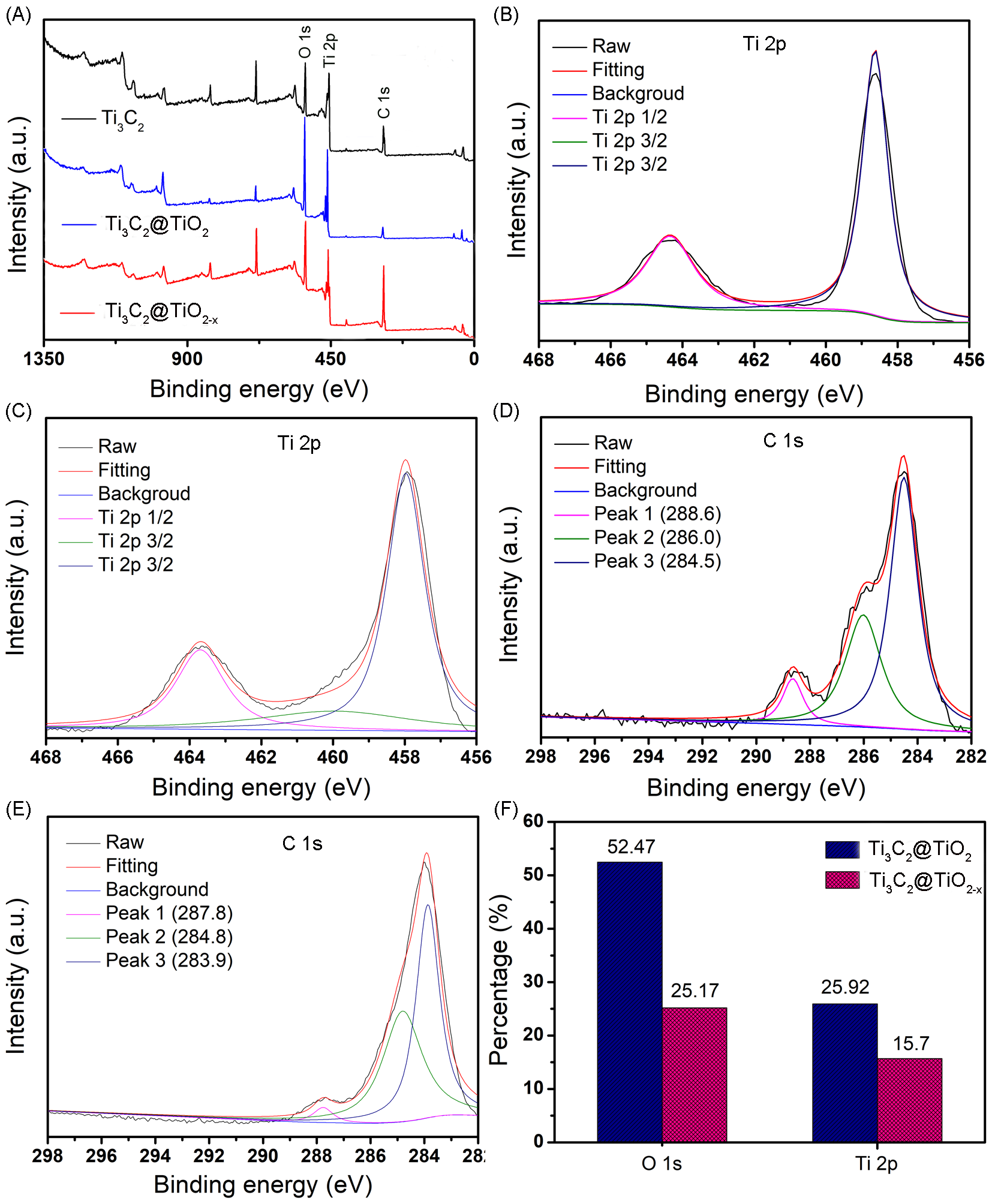


Figure S4. (A) XPS spectra of Ti_3_C_2_, Ti_3_C_2_@TiO_2_, and Ti_3_C_2_@TiO_2-x_. Ti 2p XPS spectra of (B) Ti_3_C_2_@TiO_2_ and (C) Ti_3_C_2_@TiO_2-x_. C 1s XPS spectra of (D) Ti_3_C_2_@TiO_2_ and (E) Ti_3_C_2_@TiO_2-x_. (F) Element contents of Ti_3_C_2_@TiO_2_ and Ti_3_C_2_@TiO_2-x_.


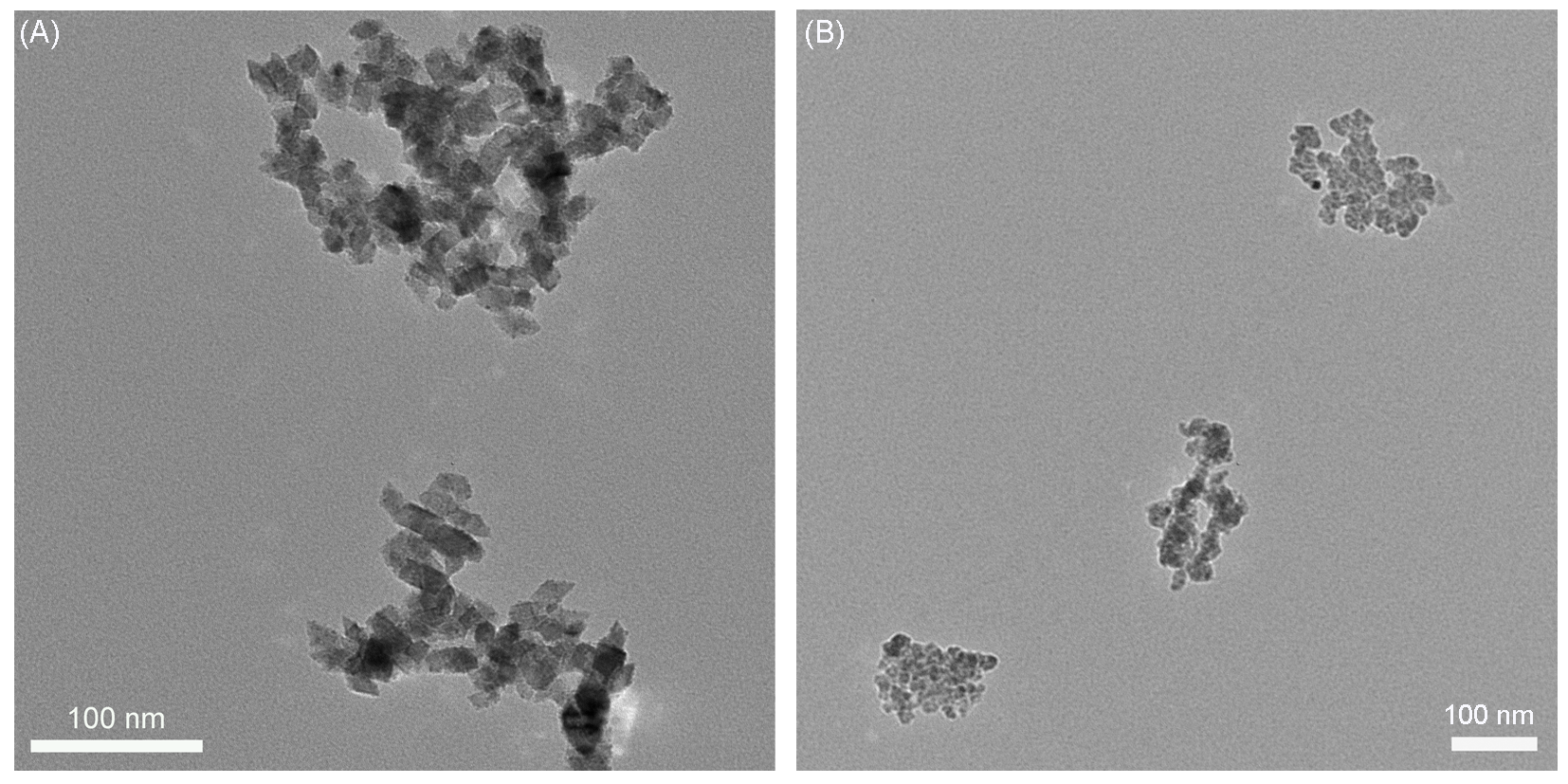


Figure S5. TEM images of (A) TTP and (B) Ti_3_C_2_@TiO_2_-PEG.


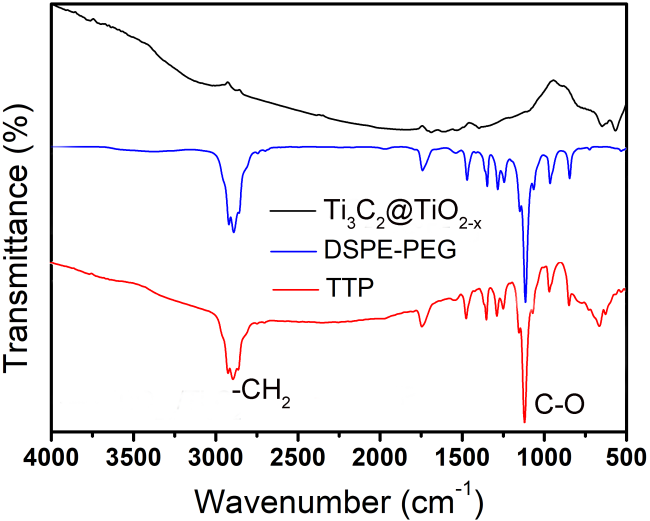


Figure S6. FT-IR spectrum of Ti_3_C_2_@TiO_2-x_, DSPE-PEG, and TTP.


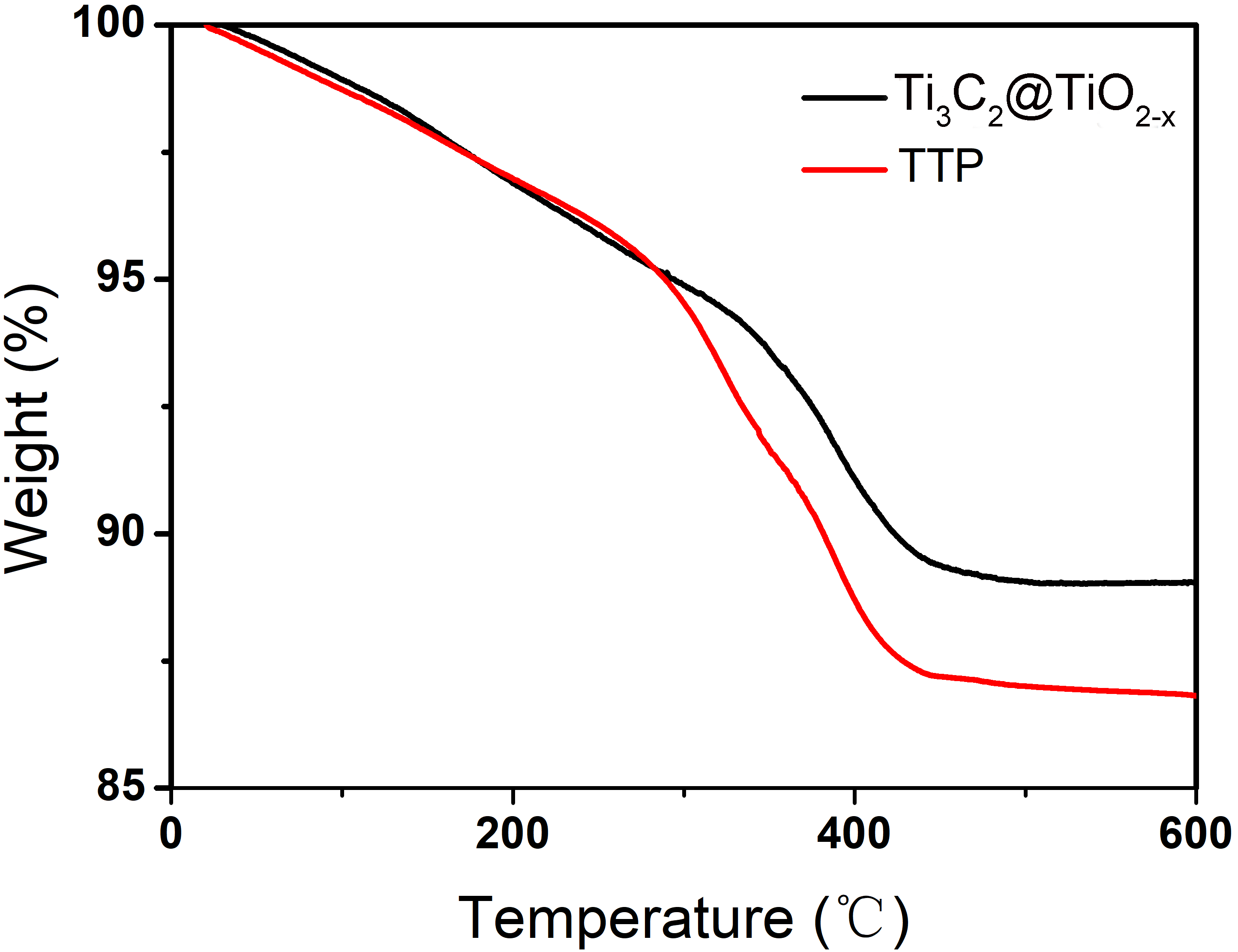


Figure S7. TGA curves of Ti_3_C_2_@TiO_2-x_ and TTP.


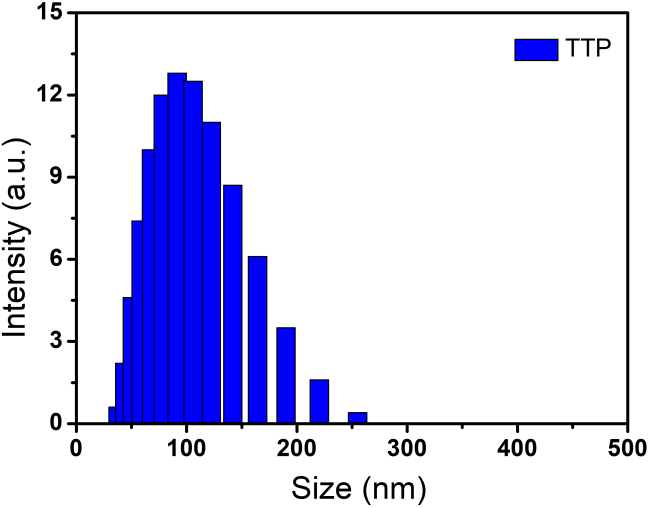


Figure S8. Hydrodynamic diameter of TTP in water as detected by DLS.


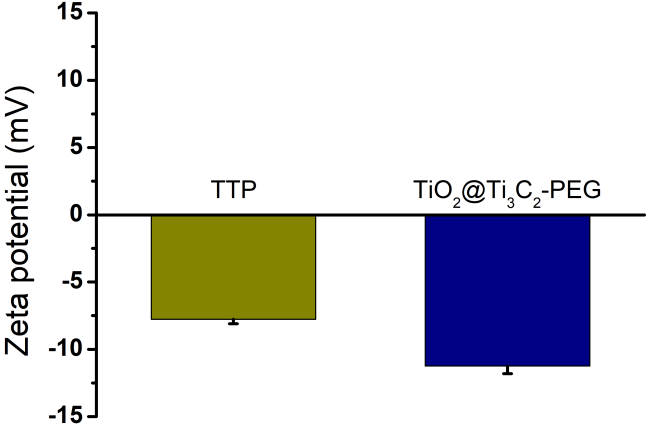


Figure S9. Zeta potential of TTP and Ti_3_C_2_@TiO_2_-PEG.


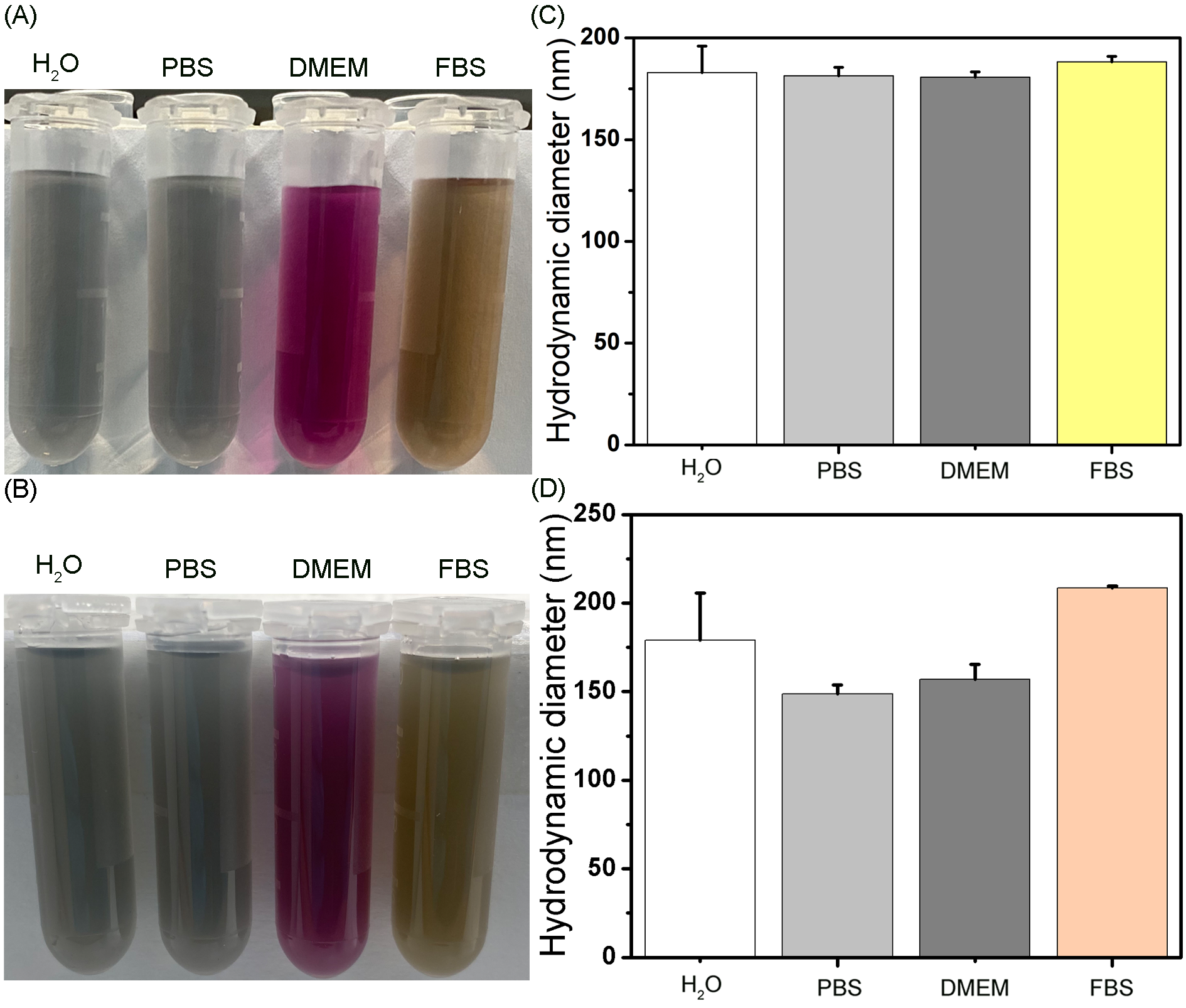


Figure S10. (A-B) Digital photographs and (C-D) hydrodynamic diameter of TTP and Ti_3_C_2_@TiO_2_-PEG in water, PBS, DMEM, and FBS solutions after 3 days.


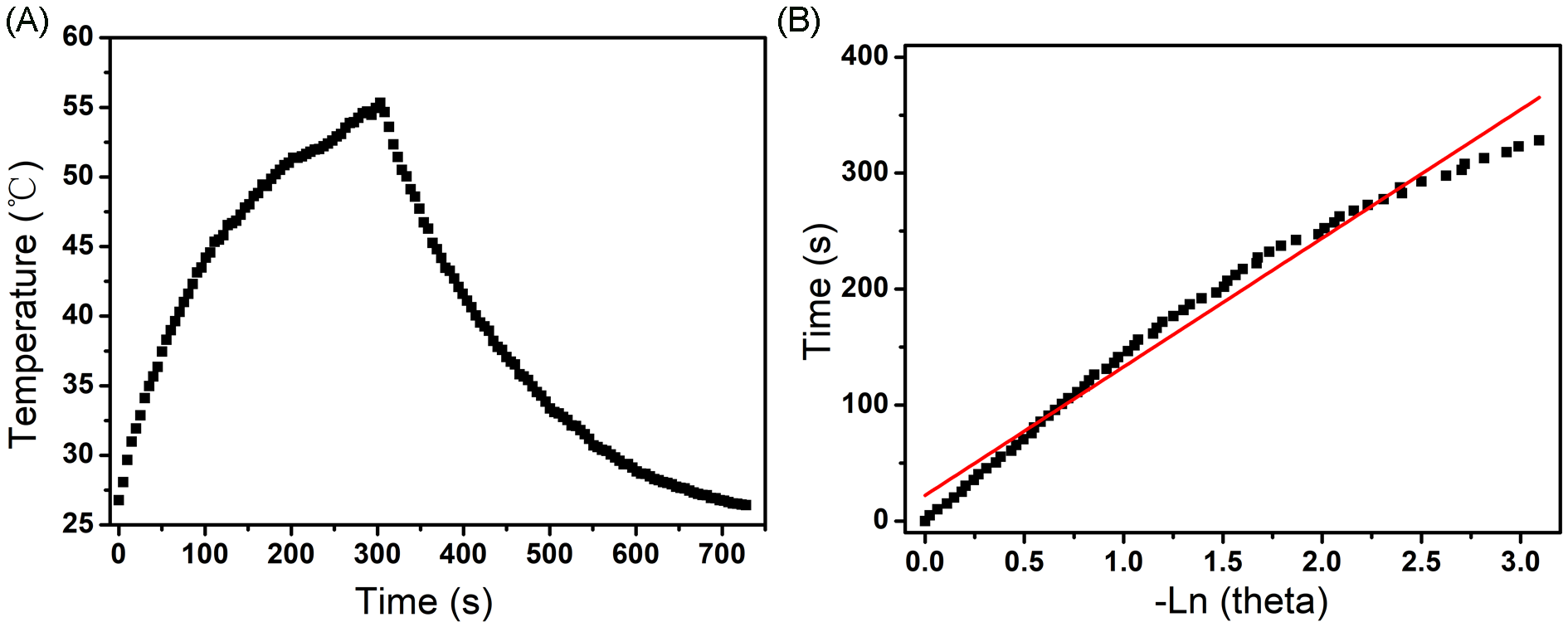


Figure S11. (A) The photothermal performance of TTP irradiated by a 1064 nm laser (0.8 W/cm^2^) irradiation for 5 min and then turn off the laser. (B) Plot of cooling time versus negative natural logarithm of the temperature driving force, which is obtained from the cooling stage. Heat dissipation time constant is determined to be *τ_s_* = 110.8 s.


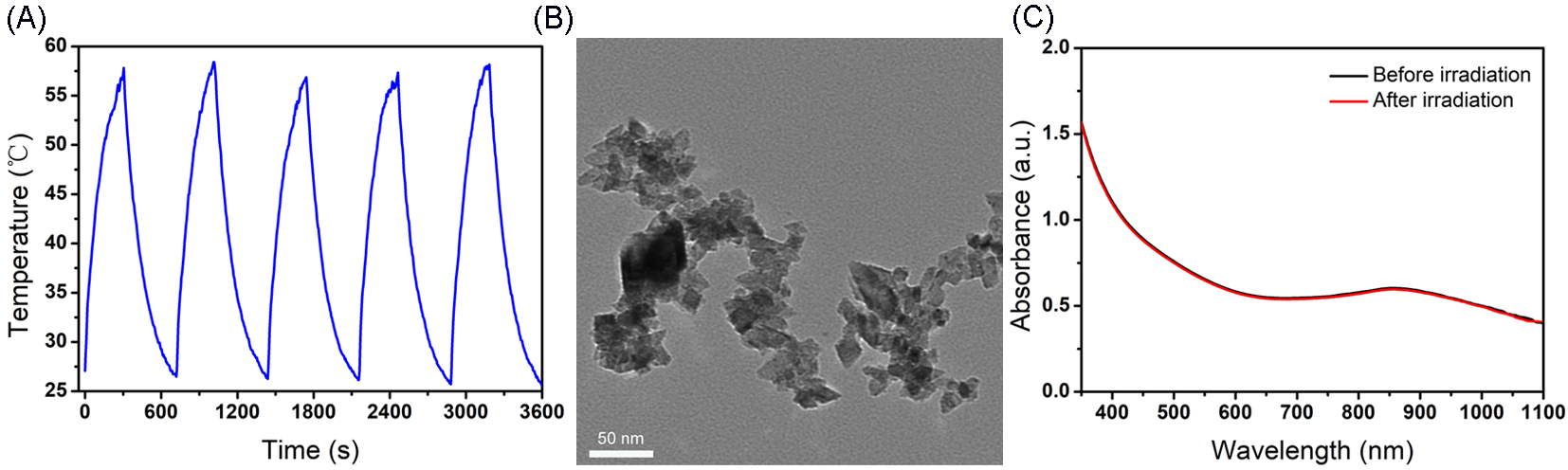


Figure S12. (A) Five-consecutive laser on/off cycles (5 min on/5 min off) to determine the photothermal stability of TTP hybrid. (B) TEM image of TTP after 1064 nm laser irradiation (0.8 W/cm^2^) for 20 min. The scale bar is 50 nm. (C) Absorbance spectrum of TTP before and after irradiation with 1064 nm laser (0.8 W/cm^2^, 20 min).


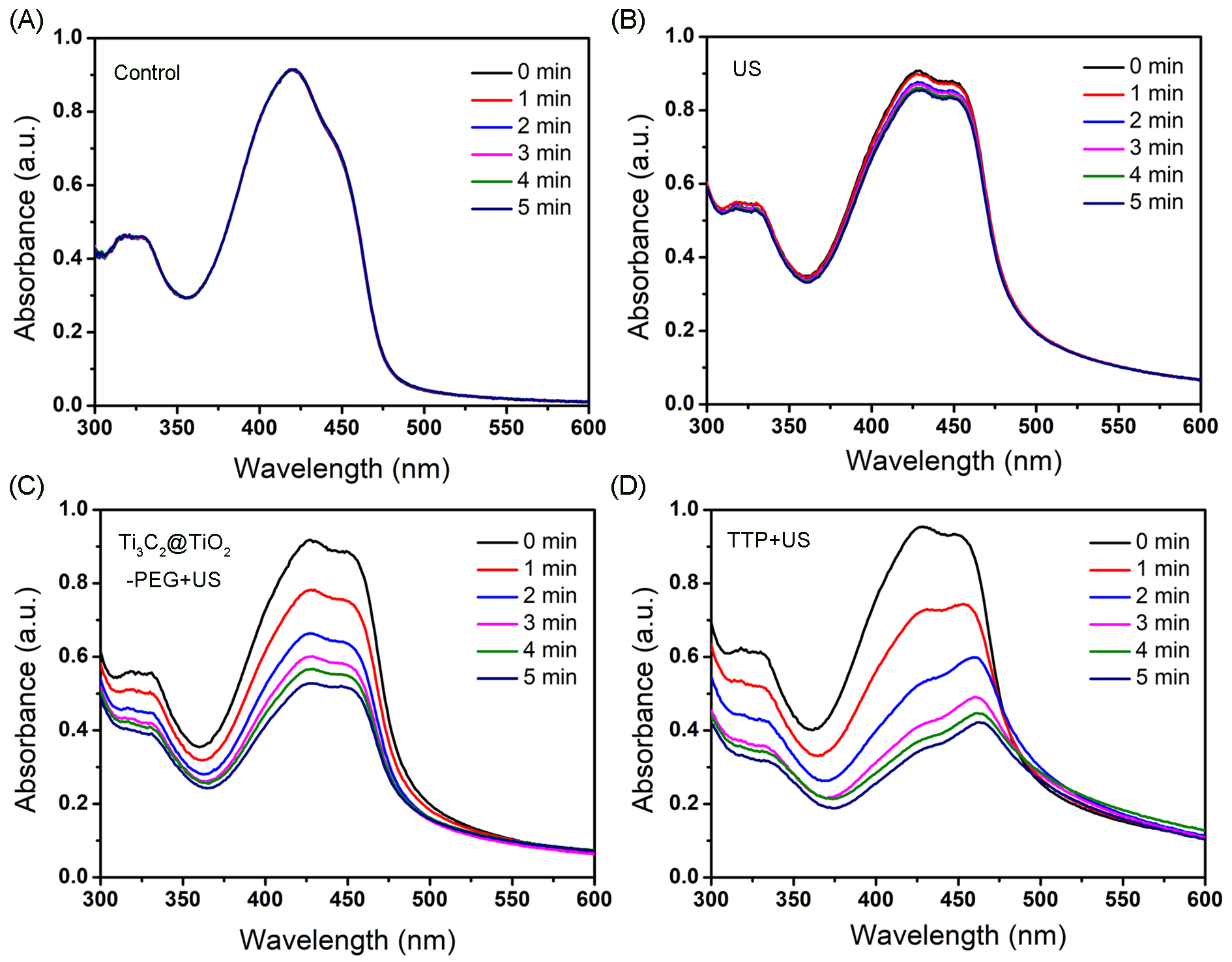


Figure S13. Changes in the absorption spectra of DPBF (25 μM) under different treatment conditions as mentioned.


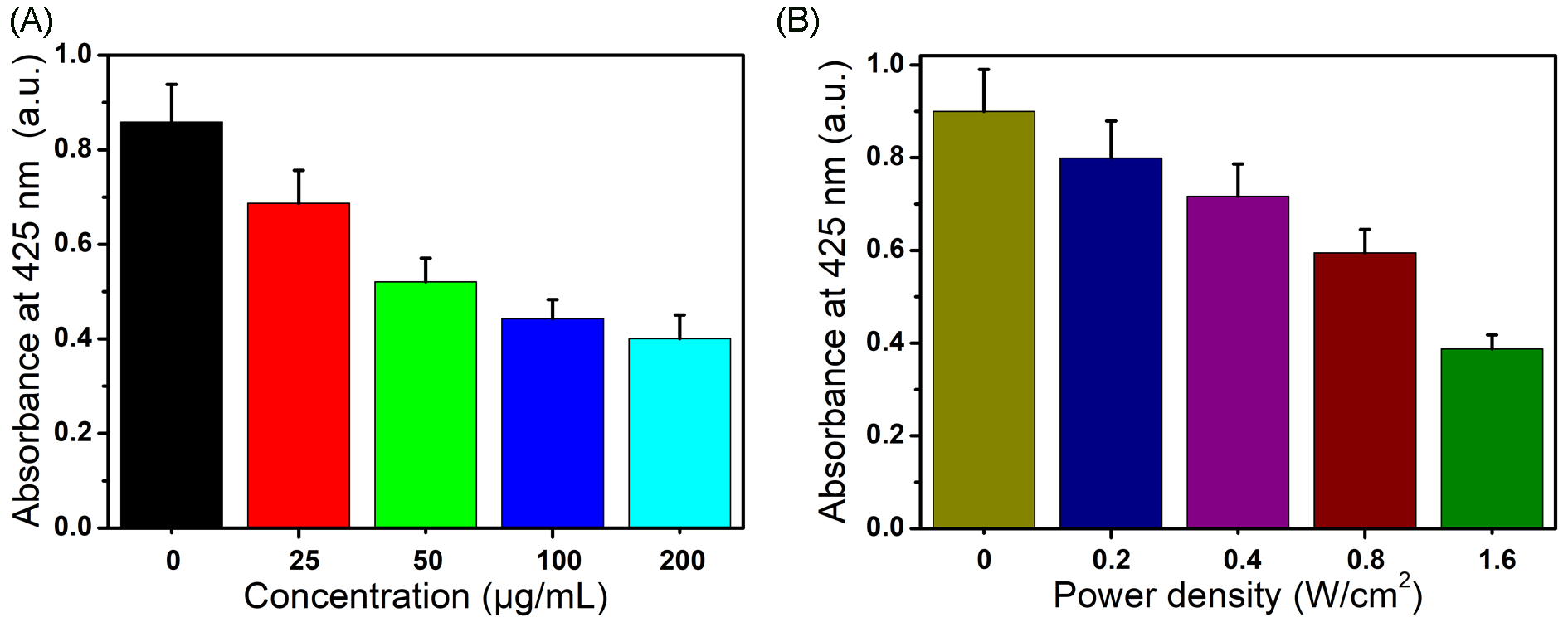


Figure S14. (A) The corresponding absorbance values at 425 nm of DPBF treated with different concentrations (0-200 μg/mL) of TTP under US irradiation (1 W/cm^2^, 1 MHz, 3 min). (B) The corresponding absorbance values at 425 nm of DPBF treated with 50 μg/mL of TTP under US irradiations with different power density (0-1.6 W/cm^2^, 1 MHz, 3 min).


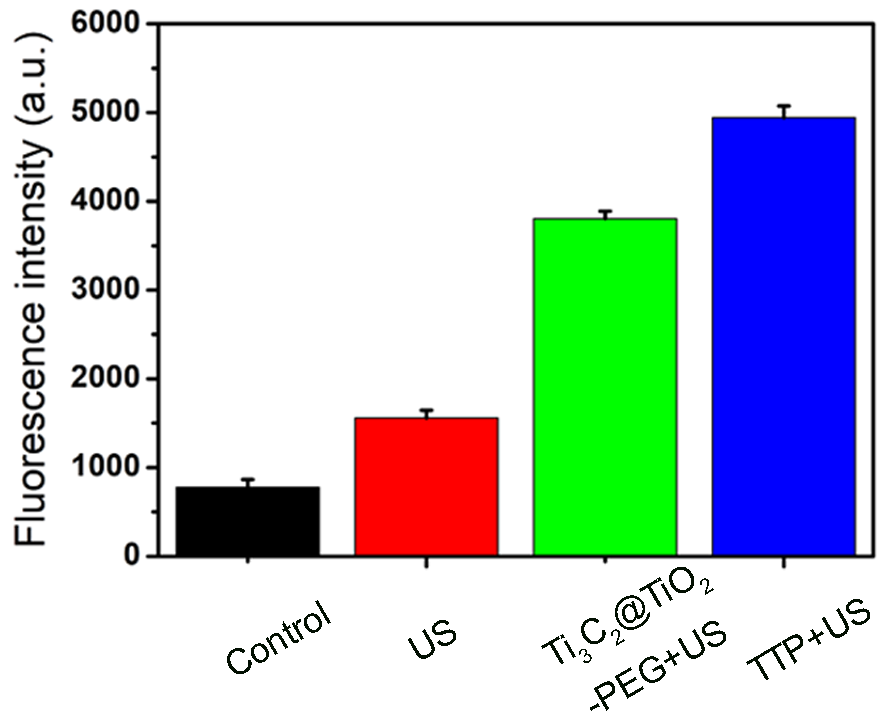


Figure S15. Fluorescence values (I) of TPA solutions at 422 nm under different conditions and US stimulation (1 W/cm^2^, 1 MHz) for 5 min.


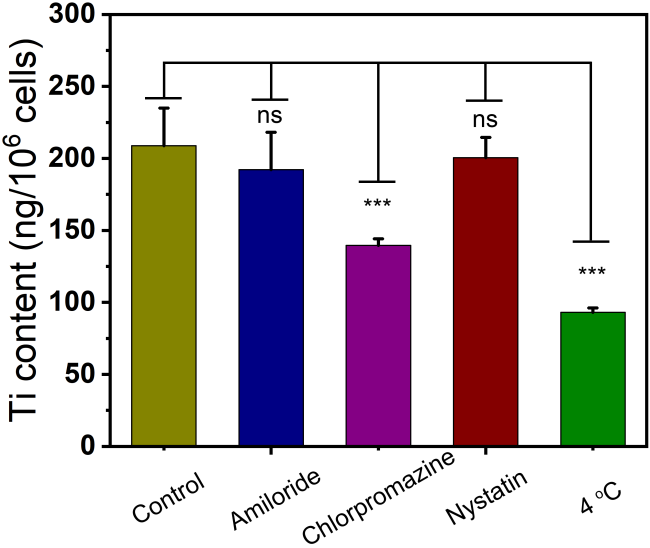


Figure S16. The cellular uptake mechanism of TTP was investigated by ICP-MS. Statistical significance compared with the control group is shown (*P < 0.05; **P < 0.01 and ***P < 0.001).


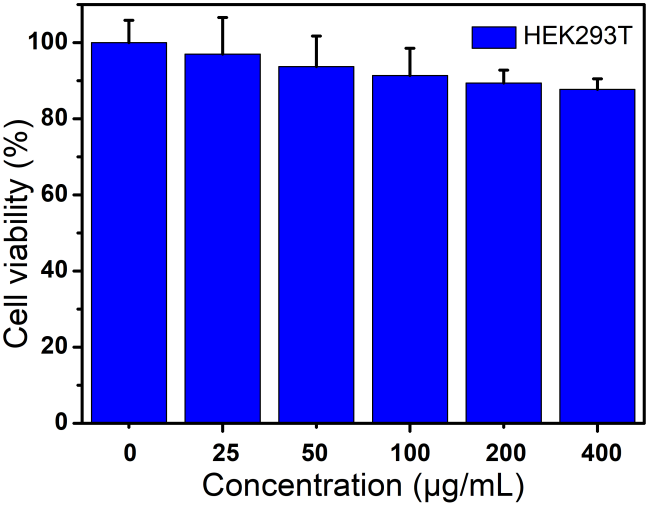


Figure S17. Cell viability of HEK293T after treated with various concentrations of TTP in the dark.


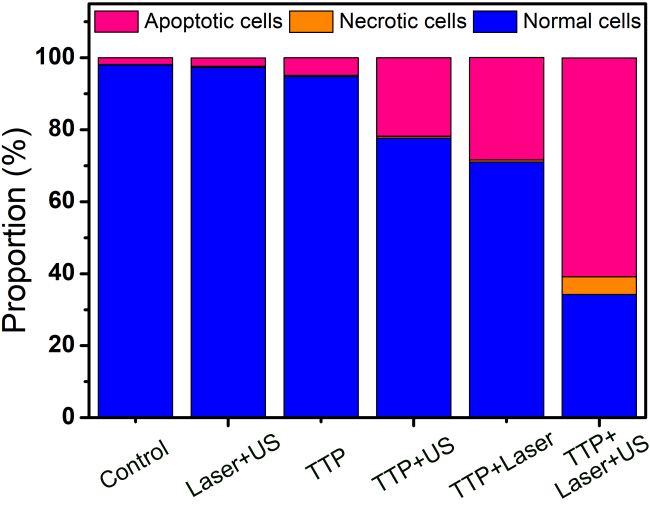


Figure S18. Quantitative assessment of 4T1 cells death (apoptosis and necrosis) and viability by flow cytometry under different treatment conditions.


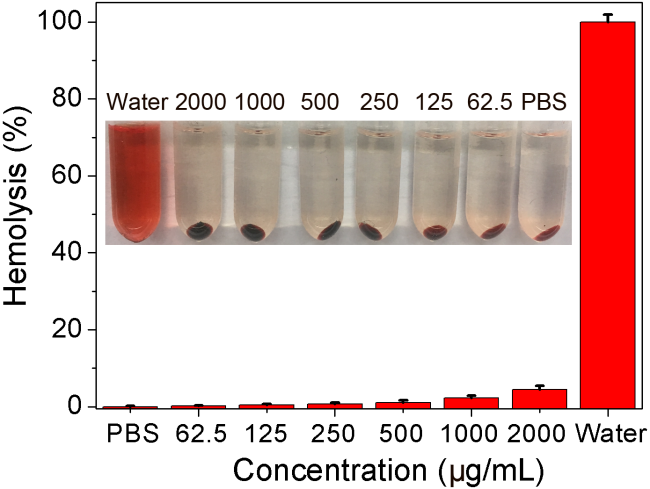


Figure S19. Hemolysis assay of TTP on red blood cells.


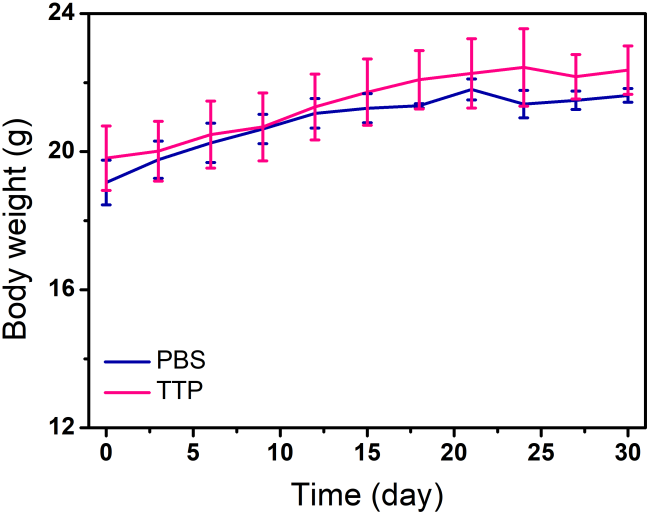


Figure S20. Changes in the body weight of normal mice after intravenous injection of PBS or TTP for one month.


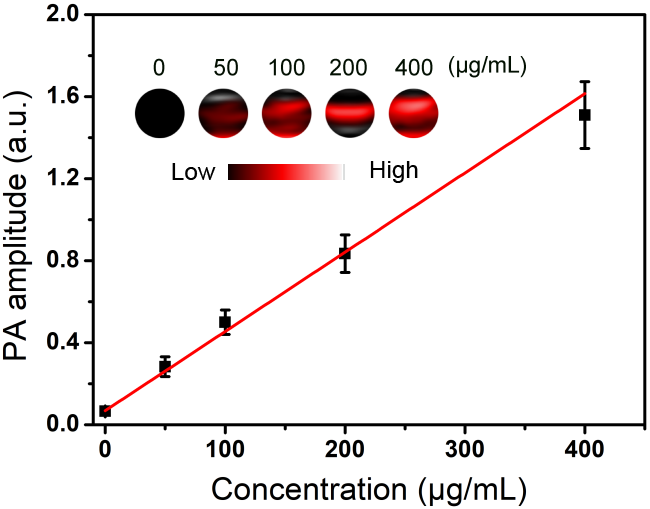


Figure S21. PA signal intensity of TTP solutions with various concentrations.


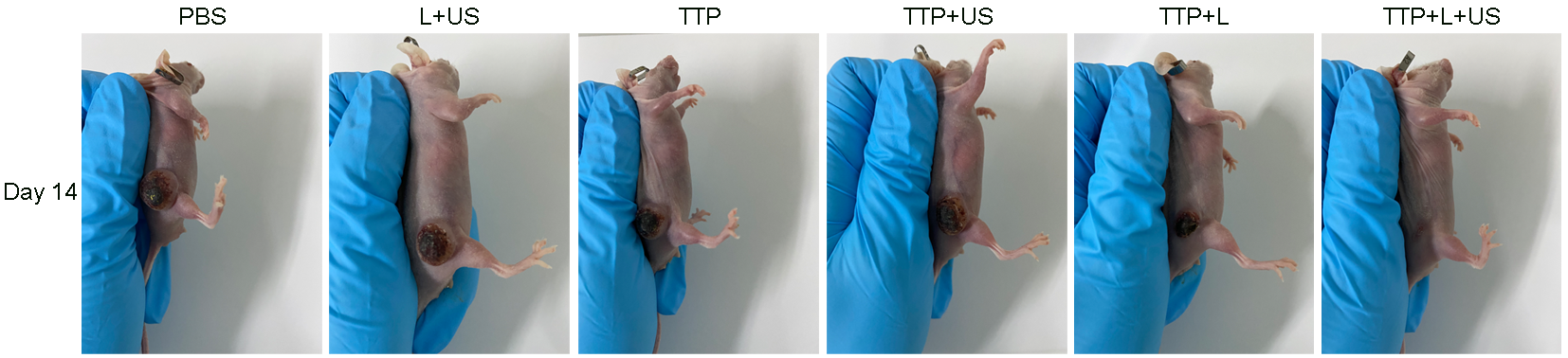


Figure S22. Digital photographs of tumor bearing mice at 14^th^ day undergo different treatment conditions as indicated.


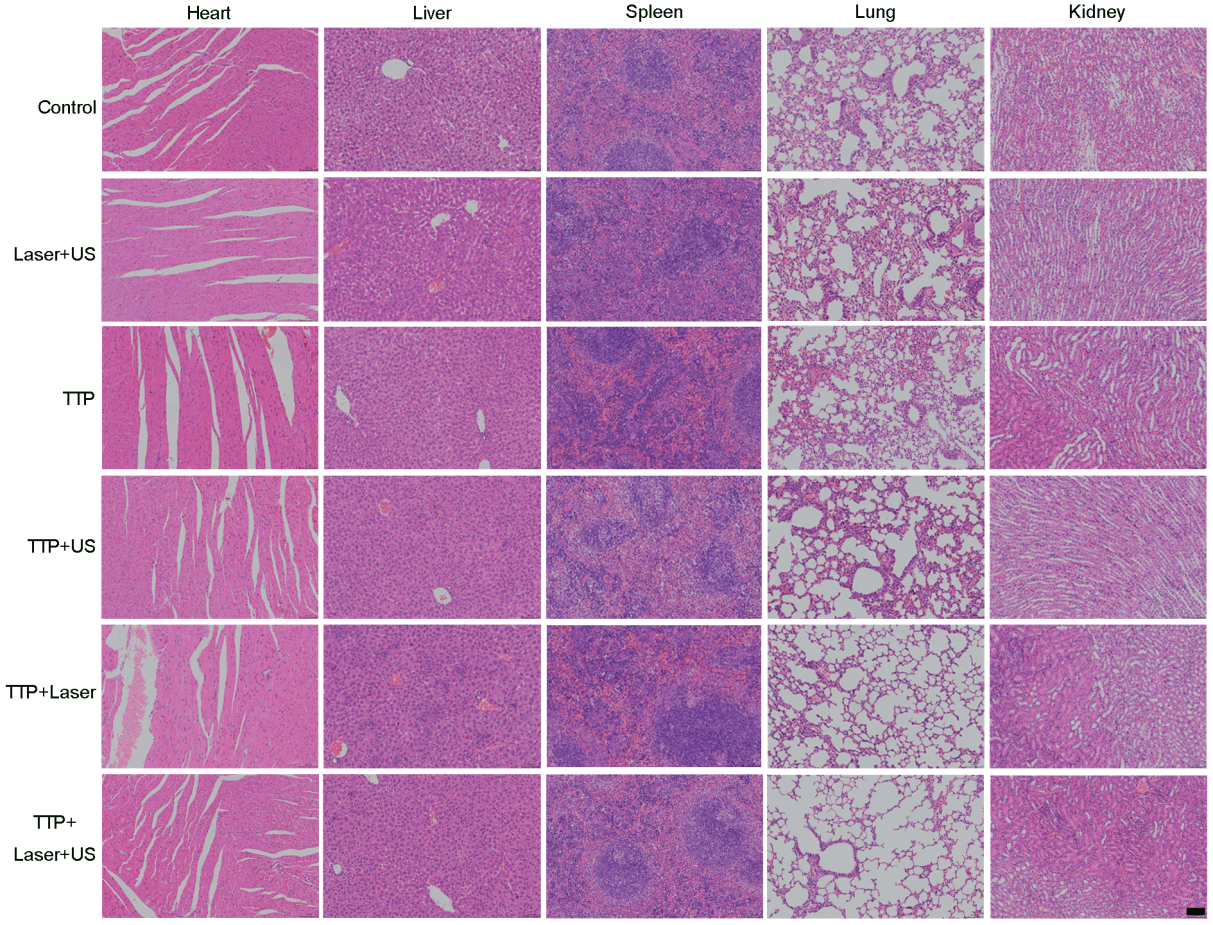


Figure S23. H&E-stained images of major organs collected at 14th day from different experimental groups as mentioned.

Reference

1. Zhang D-Y, Zheng Y, Zhang H, Yang G-G, Tan C-P, He L, et al. Folate receptor-targeted theranostic IrS_x_ nanoparticles for multimodal imaging-guided combined chemo-photothermal therapy. Nanoscale. 2018; 10: 22252-62.
